# Aberrant MET activation impairs perinuclear actin cap organization with YAP1 cytosolic relocation

**DOI:** 10.1038/s42003-023-05411-y

**Published:** 2023-10-14

**Authors:** Michela Sgarzi, Martina Mazzeschi, Spartaco Santi, Elisa Montacci, Tito Panciera, Enea Ferlizza, Cinzia Girone, Alessandra Morselli, Valerio Gelfo, Rikke Sofie Kuhre, Carola Cavallo, Sabrina Valente, Gianandrea Pasquinelli, Balazs Győrffy, Gabriele D’Uva, Donatella Romaniello, Mattia Lauriola

**Affiliations:** 1 IRCCS Azienda Ospedaliero-Universitaria di Bologna, Bologna, Italy; 2 Department of Medical and Surgical Sciences (DIMEC), University of Bologna, Bologna, Italy; 3 Institute of Molecular Genetics, National Research Council of Italy, Bologna, Italy; 4 IRCCS-Institute Orthopaedic Rizzoli, Bologna, Italy; 5 Department of Molecular Medicine, University of Padua, Padua, Italy; 6 Laboratory of Preclinical Studies for Regenerative Medicine of the Musculoskeletal System (RAMSES), (IRCCS) Istituto Ortopedico Rizzoli, Bologna, Italy; 7 Semmelweis University Dept. of Bioinformatics and 2nd Dept. Of Pediatrics, Budapest, Hungary; 8 TTK Cancer Biomarker Research Group, Institute of Enzymology, Budapest, Hungary; 9 Centre for Applied Biomedical Research (CRBA), Bologna University Hospital Authority St. Orsola -Malpighi Polyclinic, Bologna, Italy

**Keywords:** Cell biology, Molecular biology

## Abstract

Little is known about the signaling network responsible for the organization of the perinuclear actin cap, a recently identified structure holding unique roles in the regulation of nuclear shape and cell directionality. In cancer cells expressing a constitutively active MET, we show a rearrangement of the actin cap filaments, which crash into perinuclear patches associated with spherical nuclei, meandering cell motility and inactivation of the mechano-transducer YAP1. MET ablation is sufficient to reactivate YAP1 and restore the cap, leading to enhanced directionality and flattened nuclei. Consistently, the introduction of a hyperactive MET in normal epithelial cells, enhances nuclear height and alters the cap organization, as also confirmed by TEM analysis. Finally, the constitutively active YAP1 mutant YAP5SA is able to overcome the effects of oncogenic MET. Overall, our work describes a signaling axis empowering MET-mediated YAP1 dampening and actin cap misalignment, with implications for nuclear shape and cell motility.

## Abstract (web summary)

Analysis of actin dynamics in cancer reveals that MET hyperactivation drives perinuclear actin cap disruption by YAP1 inhibition, inducing nuclear expansion, meandering cell motility and collapse of the apical microvilli into actin-rich patches.

## Introduction

Nuclear irregularities have pride of place among all the histological hallmarks of cancer, having important implications in terms of cell motility during metastatic spread^1^ as well as chromatin arrangement and consequently gene expression^2^.

Over the years, a large body of reports identified a physical connection between the nuclear envelope and the surrounding cytoskeleton, highlighting a major role of actin microfilaments in the determination of nuclear architecture^3^. The so-called perinuclear actin cap, derived from the alignment of apical acto-myosin bundles, has been identified as the main player in the cytoskeletal regulation of nuclear shape^4^. Indeed, the perinuclear actin fibers represent the only subcellular structures in direct contact with both the cell periphery, through specific actin-cap associated focal adhesions (ACAFAs)^5^, and the nuclear scaffold, through the Linker of Nucleoskeleton and Cytoskeleton (LINC) protein supercomplex^6,7^. Thus, the actin cap displays the capability to integrate mechanical cues from the extracellular environment to finely modulate nuclear morphology^7^. Moreover, the alternance between actin cap dissolution and formation has been shown to subtly tune cell motility in cultured fibroblasts, polarizing cell motion in the direction of actin cap fibers for relatively long persistent movements by preventing nuclear rotation^8^.

In this perspective, a defective assembly of the actin cap has been suggested to be directly involved in the oncogenic transformation and cancer progression, by influencing both cell migration, nuclear shape regulation and cell mechanosensing^9^. Perinuclear actin fibers have been observed to be misaligned or completely absent in cell models of laminopathies, where the lack of a properly organized nuclear lamina due to lamin A/C mutations intrinsically prevents actin cap’s anchoring to the nucleoskeleton. It has also been recently reported that perinuclear actin fibers are often disrupted in several cancer cell models, even in presence of properly assembled conventional basal stress fibers^9^. Nonetheless, the mechanism that leads to a defective perinuclear actin arrangement in cancer cells is still unknown. Likewise, how oncogenic transformation itself can produce nuclear structural modifications is unclear, putting the perinuclear actin cap at the top of the list of putative mediators responsible for cancer-related nuclear abnormalities.

Oncogenic receptor tyrosine kinases (RTKs) are known to control actin remodeling by influencing, for example, the formation of membrane ruffles, actin-based structures involved in cell spreading (lamellipodia) or three-dimensional migration and macropinocytosis (circular ruffles)^10,11^. Among others, the hepatocyte growth factor (HGF) receptor (MET) has emerged as central regulator of actin cytoskeleton remodeling in the context of cell migration and scattering modulation. It has been accepted that MET activation induces a Rac1-mediated signaling cascade with actin remodeling and membrane ruffling, both by recruiting Rac1 to the plasma membrane^12^ and by endosome signaling^13^. Interestingly, MET is able to activate Rac1 from different endosomes, boosting an acute Rac1 signaling when localized in peripheral endosomes (PEs) and a sustained PI3K/Vav2-mediated Rac1 activation when trafficking to a perinuclear endosome (PNE)^13^. Nonetheless, the role of MET in the regulation of perinuclear actin fibers as well as the consequences in terms of nuclear shape and cancer cell signalling has not been explored thus far. Here we propose a model of an actin based RTKs control over the nuclear structure, which could represent a previously undefined oncogenic mechanism.

By employing a colorectal cancer cell line naturally expressing an altered constitutively active form of MET, we found that MET induces an overall dismantling of perinuclear actin cap fibers, which collapse into large actin aggregates (actin patches) leading to expanded nuclear architecture, increased cell height and unpolarized cell motility. Strikingly, the knock-out (KO) of MET gene was sufficient to restore the proper actin filaments arrangement, with perfectly aligned perinuclear actin cap bundles detected in the apical plane and, consequently, a flattened cell phenotype and increased directionality of cell movements. Interestingly, MET silencing also triggered an impressive nuclear relocation and activation of YAP1. Finally, by experimentally introducing a constitutively active form of MET in a normal epithelial cell line, we demonstrated that MET activation alone was sufficient to prevent the proper alignment of the actin cap fibers and to dampen YAP1 signalling. Conversely, the phenotype resulting from the constitutive activation of nuclear YAP by YAP5SA modification blunted the effects of oncogenic MET and finely restored the proper alignment of the actin cap fibers.

## Results

### MET ablation affects cytoskeleton organization and nuclear 3D architecture

**Fig. 1:**
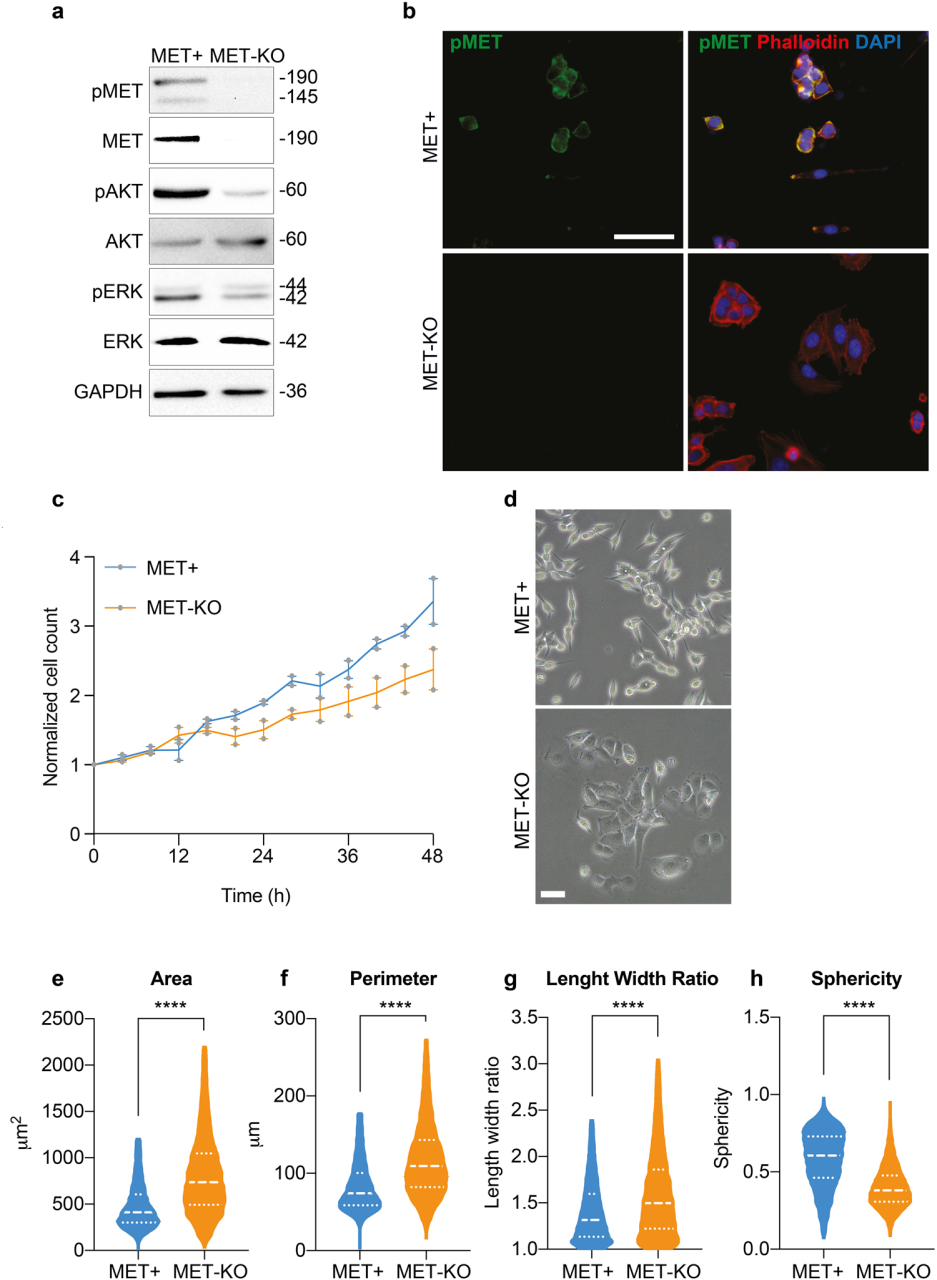
MET silencing in MET-dependent cells triggers striking morphological changes. **a** Protein analysis of MET signaling pathway in LoVo control (MET + ) and LoVo MET-KO cells. GAPDH was used as loading control. **b** LoVo MET+ and MET-KO stained with pMET (green) and Phalloidin (red) and counterstained with DAPI (blue). Scale bar: 50 μm. **c** Evaluation of cell proliferation over a time-lapse of 48 h in LoVo MET+ and MET-KO by means of Phasefocus LiveCyte^TM^ platform. Pictures were taken every 4 h. Data were normalized on cell count at T0 and shown as mean ± SEM between two independent experiments. A total of 800 cells was analyzed in each experiment. **d** LoVo MET+ and MET-KO morphology visualized with phase contrast microscopy. Scale bar: 100 μm. **e**–**h** Measurement of cell area, perimeter, length/width ratio and sphericity of LoVo MET+ and MET-KO in a 48 h time-lapse performed with Phasefocus LiveCyte^TM^. Pictures were taken every 4 h. Median and quartiles distribution are plotted. Outliers were identified and cleaned from results by means of ROUT method (Q = 1%). A total of 800 cells was analyzed. Statistic was calculated by T-test.

**Fig. 2:**
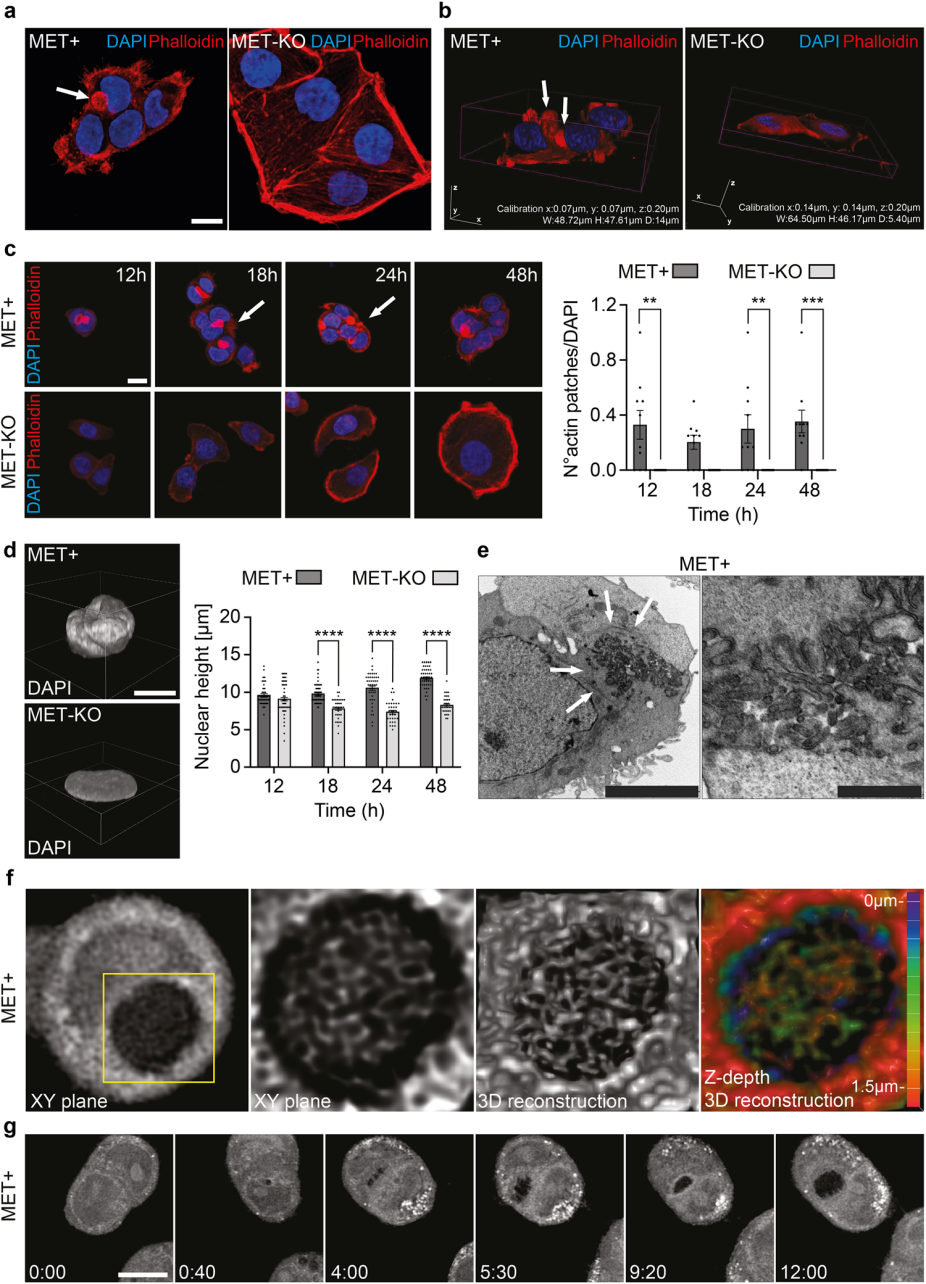
Perinuclear actin and nuclear abnormalities are present in MET hyperactivated cells and completely reverted after MET ablation. **a** Actin cytoskeleton and nucleus visualization in LoVo MET + /MET-KO fixed cells through Phalloidin (red) and DAPI (blue) staining. White arrows indicate the actin patches observed in MET+ cells. Scale bar: 20 μm. **b** Confocal 3D rendering of cells fixed and stained as in (**a**). White arrows indicate the actin patches observed in MET+ cells. **c** Nucleus and actin visualization in LoVo MET + /MET-KO cells fixed after 12/18/24/48 h from seeding. White arrows points at the actin patches. A quantification of the number of actin patches observed per field and normalized on DAPI is provided for each time point. 10 fields per condition were analyzed (181 and 135 cells for MET+ and MET-KO samples respectively). Data are reported as mean ± SEM. Statistic was calculated by Two-way ANOVA. Scale bar: 20 μm **d** Confocal 3D rendering of representative nuclei from experiment in (**c**). Nuclear height quantification was performed on 10 fields for each time point as in (**c**) by analyzing Z-stacks images. Data are shown as mean ± SEM. Statistic was calculated by Two-way ANOVA. Scale bar: 20 μm. The scale bar refers to the xy-plane. **e** Transmission electron microscopy of LoVo MET+ cells. White arrows point at actin filaments. Scale bars: left 5 μm, right 1 μm. **f** ODT and 3D reconstruction of LoVo MET+ aberrant actin patches. Z-depth coding is reported as a scale of colors. **g** ODT time lapse of LoVo MET+ cells showing actin patches generation. Pictures of cells were acquired every 10’ for 12 h. Full video is provided as Supplementary video 3. Scale bar: 10 μm.

**Fig. 3:**
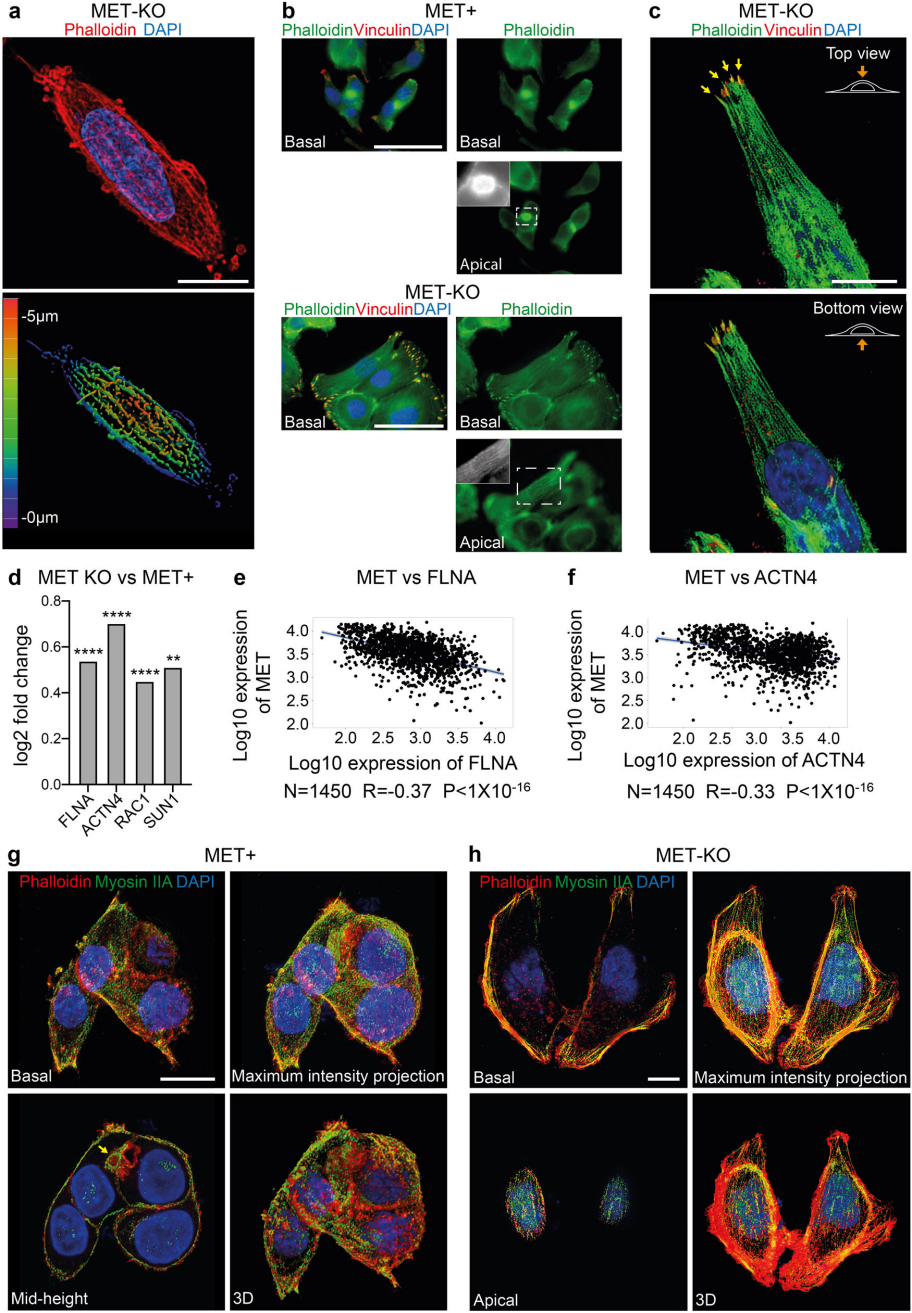
MET knock-out in MET dependent cells prompts proper organization of the perinuclear actin cap. **a** Super resolution imaging of LoVo MET-KO cells stained with Phalloidin (red) and DAPI (blue) performed with X-Light V3 – DeepSIM microscope. Z-depth coding is reported as a scale of colors in the lower panel. Scale bar: 10 μm. **b** Focal adhesions (red) and actin cytoskeleton (green) visualization through Vinculin antibodies and Phalloidin staining in LoVo MET-KO and MET+ cells. Nuclei were stained with DAPI (blue). Pictures of basal and apical plane of cells are provided. Scale bar: 50 μm **c** Top and bottom view of a MET-KO cell stained with Phalloidin (green), Vinculin (red) and DAPI (blue) and visualized in 3D reconstruction. Yellow arrows point at the Actin Cap Associated Focal Adhesions (ACAFAs). Scale bar: 10 μm. The scale bar refers to the xy-plane. **d** Log2 fold change of *filamin A* (*FLNA*), *actinin 4* (*ACTN4*), *Rac Family Small GTPase 1* (*RAC1*) and *Sad1* and *UNC84 Domain Containing 1* (*SUN1*) expression in LoVo MET-KO cells compared to LoVo MET+ cells according to RNAseq data. ****Padj < 0.0001 **Padj < 0.01. **e**, **f** Pearson correlation between the expression of MET and FLNA (**e**) or ACTN4 (**f**) in colorectal cancer according to gene arrays data available on GEO. R and *P*-value are reported. **g**, **h** Non-muscle myosin IIA (green), actin cytoskeleton (Phalloidin – red) and nuclei (DAPI – blue) staining in MET-KO and MET+ cells. Pictures from different planes of section, as well as maximum intensity projection and 3D rendering are provided. Scale bar: 10 μm.

LoVo cells encode for an aberrant uncleaved MET receptor of 190 kDa constitutively phosphorylated independently from the ligand^14^. This alteration occurs at a post-translational level, and MET gene is neither mutated nor amplified in these cells^14^. Thus, LoVo cell line represents a unique and ideal model to study MET-driven phenotypical alterations in cancer cells. By means of CRISPR/Cas9 technology^15^, we performed a MET gene KO. Successful MET ablation was confirmed by protein analyses both by western blot (Fig. 1a) and immunofluorescence (Fig. 1b). In line, the activation of MET downstream effectors, AKT and ERK appeared downregulated in MET-KO cells (Fig. 1a). Furthermore, MET-KO ablation reduced cell proliferation rate (Fig. 1c) and triggered an impressive modification in the cell phenotype: indeed, the typical fibroblastic-like feature of MET aberrant cells was clearly reverted upon ablation, turning the cells into a squamous epithelial shape with enhanced cell boundaries (Fig. 1d). The Ptychographic quantitative phase imaging technology of Phasefocus Livecyte^TM^ system allowed to precisely assess in living cells several morphometric parameters by measuring the main phenotypical changes derived from MET silencing. First, we detected a statistically significant increment of the cellular area and the perimeter (Fig. 1e, f) in MET-KO compared to MET aberrant (MET + ) cells. In addition, the morphological differences triggered by MET ablation were confirmed by a remarkable increase in length-width ratio (Fig. 1g), suggesting an elongated cell shape associated with a strong reduction in cells’ sphericity (Fig. 1h). Prompted by the remarkable morphological changes observed, we further investigated the actin microfilaments organization of the MET-KO cells in comparison with the control cells, where MET signaling is constitutively activated. Using both 2D standard immunofluorescence (Fig. 2a) and 3D reconstruction from confocal imaging (Fig. 2b), we recorded a precise orientation of the actin cytoskeleton. In detail, the 3D rendering revealed that MET-activated cells bear aberrant actin structures, located close to the nucleus, associated with a well-defined rounded nuclear architecture, and distributed over the entire cell height (Fig. 2a, b, white arrows). In normal conditions, nuclei are usually covered and stretched by bundles of actomyosin, which connects the nuclear envelope to the cytoskeleton. In MET+ cells, the derailed actin fibers alignment leads to the coalescing of the fibers in the apical plane, by forming actin structures, to which we refer to as *actin-patches*. Notably, MET KO cells reverted to a quasi-normal actin filament fibers configuration (Fig. 2a–c) with the actin-patches structures completely disappearing, while remaining stable over time in aberrant MET+ cells (Fig. 2c). Interestingly, the nuclear height impressively decreased upon MET ablation, and these changes were evident already after 24 h from seeding (Fig. 2d). On average, after 48 h the nuclear height measured about 12 μm in MET+ positive cells, while in KO the average height was about 8 μm (Fig. 2d and quantification). Through the Operetta CLS High-content screening system we performed further quantitative analyses on a large number of cells. MET-KO cells despite having increased nuclear volume and surface area (Supplementary Fig. 1a, b), displayed decreased nucleus sphericity and height (Supplementary Fig. 1c, d), with a markedly prominent nucleus footprint area (Supplementary Fig. 1e) consistent with a flat squamous morphology. Aiming to confirm these observations in additional MET+ dependent cellular models, we decided to evaluate actin cytoskeleton morphology in human gastric carcinoma GTL16 cells, characterized by MET activation due to > 10 fold MET gene amplification and overexpression, in absence of activating mutations^16^. Intriguingly, around 20% of analyzed cells showed actin aberrations like the actin patches detected in LoVo cell line (Supplementary Fig. 2a, b - white arrows), associated with spherical nuclei (Supplementary Fig. 2c). We further employed ultrastructural imaging by transmission electron microscopy (TEM) to visualize the aberrant actin patches in MET+ cells. Intriguingly, TEM analysis highlighted abnormal groups of internalized microvilli-like structures (Fig. 2e, detail on the right) collected inside rounded and disorganized actin-based thick bundles (Fig. 2e, left panel, white arrows). We propose that the perinuclear microfilaments, by coalescing at the cell surface, could induce a global invagination of the cell membrane and consequent internalization of the apical microvilli. Next, we set to confirm by live-cell imaging that these actin patches were not caused by artifactual fixation of actin bundles. We employed a retroviral vector encoding for the LifeAct peptide, which is able to stain actin microfilaments in living cells without impairing actin polymerization^17^, conjugated with mCherry fluorophore. We monitored overtime the actin dynamics of MET aberrant cells stably expressing mCherry-LifeAct. As reported in Supplementary videos 1 and 2, LoVo-LifeAct replicated the actin-patches aberrations observed in fixed cells, and by monitoring them over time, we recorded structures highly dynamic and continuously rotating around the nucleus (Supplementary Fig. 3a, Supplementary videos 1 and 2). Better resolved images of the aberrant actin patches were obtained in label-free conditions by means of Optical Diffraction Tomography (ODT), which detects variations between the refractory indexes (RI) of different cell compartments. With ODT imaging, the actin patches appeared as circular structures with a higher RI on the edges and a lower RI inside, where a jagged and reticular pattern suggests the presence of chaotic actin filaments (Fig. 2f, Supplementary Fig. 3b). The overlap between LifeAct and Phalloidin signal with ODT detection was confirmed in fixed cells as reported in Supplementary Fig. 3c, d (yellow arrows). Moreover, in living MET+ aberrant cells ODT time-lapse imaging recorded the progressive development of the actin patches, that reached the mature structure after 12 h (Fig. 2g, Supplementary video 3). Intriguingly, during cell division, actin-patches showed asymmetric distribution and only one of the two daughter cells inherits the aberrant actin-patches (Supplementary video 4). Finally, the absence of similar actin abnormalities in the MET-KO model was validated also with ODT technology (Supplementary video 5). Overall, ODT and LifeAct staining confirmed the presence of an abnormal and steadily moving actin-based formation, namely actin-patches, in cells with MET+ sustained activation. These structures maintain a physical connection with the nuclear envelope, with a consequent asymmetric confinement during cell division.

### MET inactivation is required for the appropriate perinuclear actin cap assembly

The cytoskeletal tensions provided by the aligned perinuclear actin cap in interphase cells have been reported to compress the nucleus and to maintain a flat phenotype^4^. Therefore, we hypothesized that MET-driven alterations may associate to a misalignment of the perinuclear actin fibers, resulting in increased nuclear height. First, using a super-resolution microscope, we were able to observe the restored alignment of the actin cap fibers, in MET-KO cells (Fig. 3a). These fibers now cover the entire nuclear height on the apical plane, as appreciable in the 3D rendering of the phalloidin staining in Fig. 3a. Next, we applied vinculin staining to detect the terminal edges of the actin fibers, ending into the focal adhesions (FAs). MET+ aberrant cells displayed poorly organized FAs, most likely due to the lack of a proper alignment of the actin fibers, which fail to reach the periphery of the cells and bind vinculin adhesion protein (Fig. 3b). On the contrary, MET ablation restored the actin microfilaments, with properly aligned actin bundles detected in the apical plane (Fig. 3b and black and white insert). Also, a remarkably well organization of the FAs was easily detectable by immunofluorescence in the basal plane of KO cells. Indeed, the single fibers of the cap cover the apical plane by ending at the cellular edge, where they bind with the actin cap-associated focal adhesion (ACAFAs) (arrows in Fig. 3c). Consistently, genes of the actin cap superfamily, encoding for structural proteins such as *filamin A* (*FLNA*) and *alpha-actinin 4* (*ACTN4*), together with the master regulator of actin cytoskeleton reorganization (*RAC1*), and the LINC complex protein *SUN1* appeared upregulated in MET KO compared to MET positive cells (Fig. 3d). So far, these results hint that MET activation may represent a negative regulator for the perinuclear actin cap genes. Following this rationale, we set to interrogate a dataset of gene array data collected from 1450 CRC patients^18^. Interestingly, we outlined a significant negative correlation between the amount of MET and the expression of both FLNA and ACTN4 (R = − 0.37 and −0.33 respectively) (Fig. 3e, f). The functionality of the perinuclear actin stress fibers is ensured by the association with non-muscle myosin IIA units, which confer contractility to the apical fibers^4^. In MET+ aberrant cells the actin-rich patches appeared surrounded by myosin IIA, which formed a ring myosin decorated structure, likely resulting from an altered organization of the actin cap bundles, which collapsed into these spots enriched for actin (Fig. 3g mid-height). On the contrary, in KO cells the full-length of the perinuclear actin filaments was associated with myosin IIA, indicating a fully functional contractile actin cap (Fig. 3h). To further prove that MET is the main mediator of actin cap cytoskeletal changes, we performed a rescue experiment, which restored the activation of MET by introducing a fusion protein of constitutively active MET, namely TPR-MET^19^ (Supplementary Fig. 4a). MET rescue strikingly returned the phenotype of LoVo cells (Supplementary Fig. 4b), resulting in a boosted proliferation rate (Supplementary Fig. 4c), decreased cell area (Supplementary Fig. 4d) and perimeter (Supplementary Fig. 4e). Most importantly, we recorded an increased vertical expansion of the cells, quantified as cell sphericity (Supplementary Fig. 4f) and thickness (Supplementary Fig. 4g), in accordance with a decreased alignment of the actin cap fibers, which induces nuclear and cell stretching and flattening. Furthermore, when we analyzed the panel of actin cap assembly mediators, namely *FLNA*, *ACTN4*, *SUN1* and *RAC1*, we recorded a significant decrease of their mRNA expression upon MET+ reintroduction (Supplementary Fig. 4h). Next, we employed Latrunculin B (LatB), an actin polymerization inhibitor that was reported^4^ to selectively block the formation of highly dynamic actin structures at low doses, including the actin cap filaments, while leaving the more stable basal fibers unaffected. We further proved that the actin bundles observed in KO cells were the actual perinuclear actin cap fibers. Indeed, in KO cells, LatB abolished actin caps in a time-dependent manner, as shown by phalloidin and myosin IIA staining (Supplementary Fig. 5a). Notably, also the actin-rich patches of MET+ aberrant cells seemed to be affected by LatB treatment (Supplementary Fig. 5b), confirming the initial observation of actin-based dynamically forming structures (about 12 h). In conclusion, MET+ constitutive activation induces a global misalignment and poor organization of actin cap bundles and FAs with the formation of perinuclear actin patches, while MET-KO ablation completely restores the alignment of the actin fibers network. Finally, to further validate that the morphological changes observed in terms of cell height and area in MET+ and MET-KO cells are a direct biological consequence of actin cap abrogation, we experimentally induced the actin cap disruption by a mixture of siRNAs against LINC complex components, namely Nesprin 1/2 and SUN1/2, (Supplementary Fig. 5c) in a normal epithelial model, MCF10A cells, which represent a useful tool for studying cell migration^20^. Here, upon siRNA transfection, we testified a complete loss of the actin cap alignment (Supplementary Fig. 5d) resulting in a mild decrease of the cell area and enhanced cell thickness (Supplementary Fig. 5e, f). Overall, these data confirm that silencing LINC complex genes, thus interfering with the actin cap, is sufficient to overcome the cell phenotype produced by loss of MET.

### MET-driven perinuclear actin aberration impairs persistent cell polarization during random motility

**Fig. 4:**
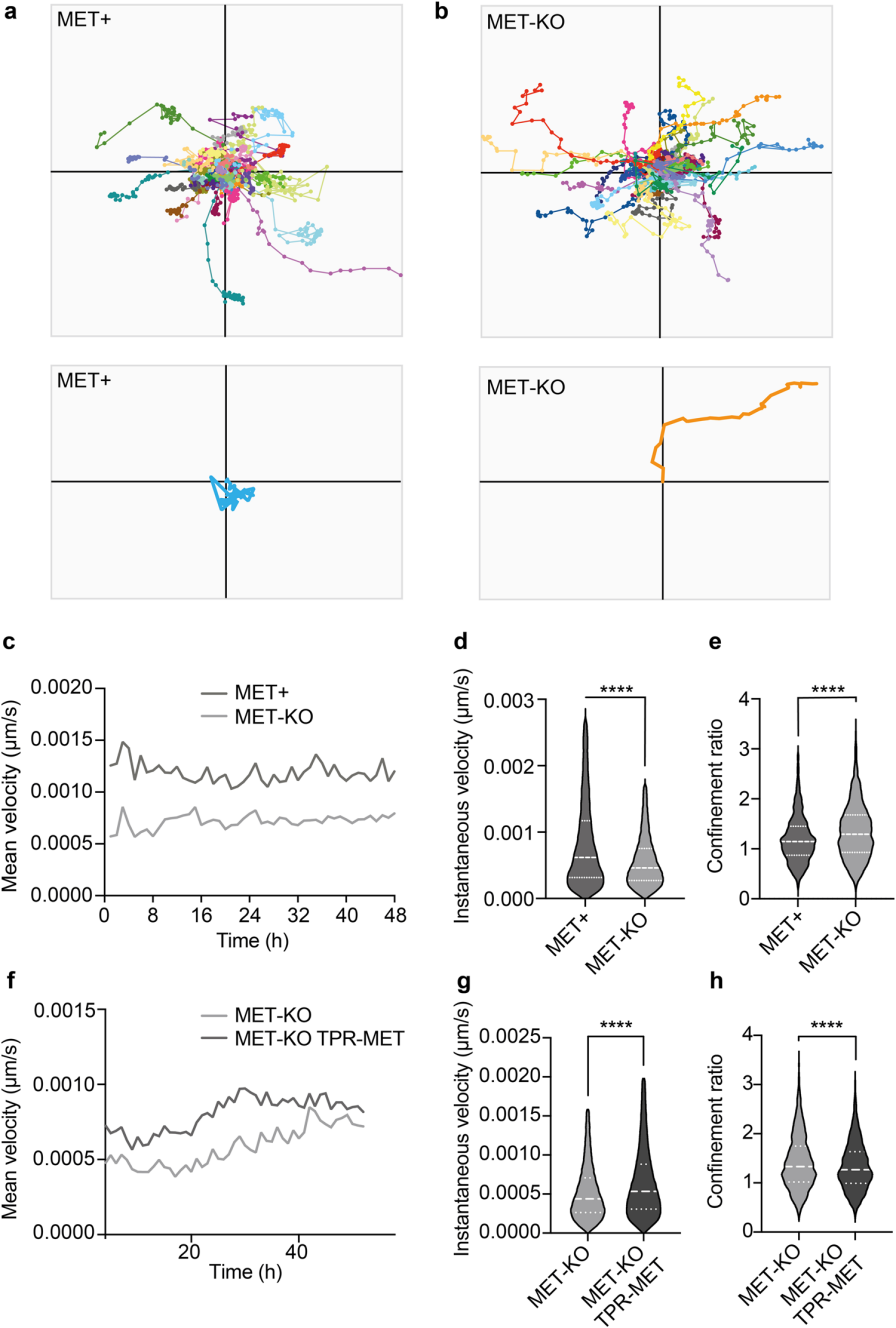
Directed and persistent cell movements are impaired in MET+ cells and restored upon MET silencing. **a**, **b** Cell tracking of LoVo MET+ and MET-KO cells monitored for 48 h with Phasefocus LiveCyte^TM^ platform. A total of 100 cells tracks are reported. Representative cell tracks were isolated and enlarged. Representative videos are reported as Supplementary videos 6, 7. **c**–**e** Charts showing mean velocity, instantaneous velocity and confinement ratio of MET+ and MET-KO cells from experiment in (**a**, **b**). Confinement ratio was computed by Phasefocus LiveCyte^TM^ software for each time frame for a track, and data are reported as the mean of these (track averaged confinement ratio). Median and quartiles distribution are reported. 800 cells were evaluated. Statistic was calculated by T-test on cleaned data according to ROUT method (Q = 1%). **f**–**h** Mean velocity, instantaneous velocity and confinement ratio of MET-KO and MET-KO TPR-MET (rescued) cells monitored over time as in (**a**, **b**). Median and quartiles distribution are reported. Statistic was calculated by T-test on cleaned data according to ROUT method (Q = 1%). 1500 cells were analyzed.

The proper organization of the actin cap fibers is required for the establishment of directionality during persistent movements in random motility settings^8^. Indeed, the cap fibers align according to the direction of cell migration for relatively long periods of time (from 30’ to 2 h), fostering nucleus translocation, and then disassemble to allow nucleus rotation and re-polarization in the direction of the next persistent move^8^. Thus, we decided to investigate cell polarization and directionality using Phasefocus LiveCyte^TM^ apparatus. Consistently with an aberrant actin cap organization, MET+ cells showed a random migration pattern characterized by scattered and meandering movements, often resulting in condensed tracks starting and ending at the same point on X-Y (Fig. 4a, Supplementary video 6). The migratory phenotype observed in MET+ cells suggests that the identified actin patches, by remaining dynamically coupled to the nuclear envelope through the LINC complex, impair polarized movements in place of random nuclear rotation. On the other hand, MET-KO cells showed well-polarized paths and perfectly calibrated persistent moves, resulting in linear and straight tracks (Fig. 4b, Supplementary video 7). In association with the different migratory behavior, MET expressing cells displayed remarkably increased velocity compared to the KO (Fig. 4c, d). Quantitatively, the different behavior of MET aberrant and KO cells can be described by the difference in the confinement ratio, which measures the straightness of a cell track^21^. Indeed, MET-KO cells displayed impressively higher confinement ratio, compatible with a directed and polarized motility, compared to the MET+ expressing cells (Fig. 4e). Interestingly, the rescue of MET hyperactivation through TPR-MET insertion in LoVo MET-KO cells was sufficient to prompt cell motility, with TPR-MET cells showing strikingly higher velocity compared to MET-KO (Fig. 4f, g, Supplementary video 8). Nonetheless, MET+ rescued cells displayed a reduced confinement ratio, confirming that MET signaling hyperactivation is per se responsible for the determination of migratory behavior (Fig. 4h, Supplementary video 8). To sum up, MET-mediated cytoskeletal alterations interfere with cell polarization during persistent random motility, by preventing the formation of a properly aligned actin cap, and most likely by favoring nuclear rotation at the expense of nuclear translocation. To confirm that these changes in cell motility were a direct consequence of the actin cap disruption, we monitored over-time the migration of normal cells (MCF10A) upon actin cap abrogation through siRNA against SUN1/2 and Nesprin 1/2. In line with our data, siRNA-treated MCF10A showed a decreased confinement ratio, although also cell velocity was reduced (Supplementary Fig. 5g, h). All together these results confirmed a driver role for MET on impairing actin cytoskeleton assembly, loss of the actin cap genes and a scattered and amoeboid motility, supporting the original hypothesis that a properly aligned actin cap may mediate polarized and straight movements.

**Fig. 5:**
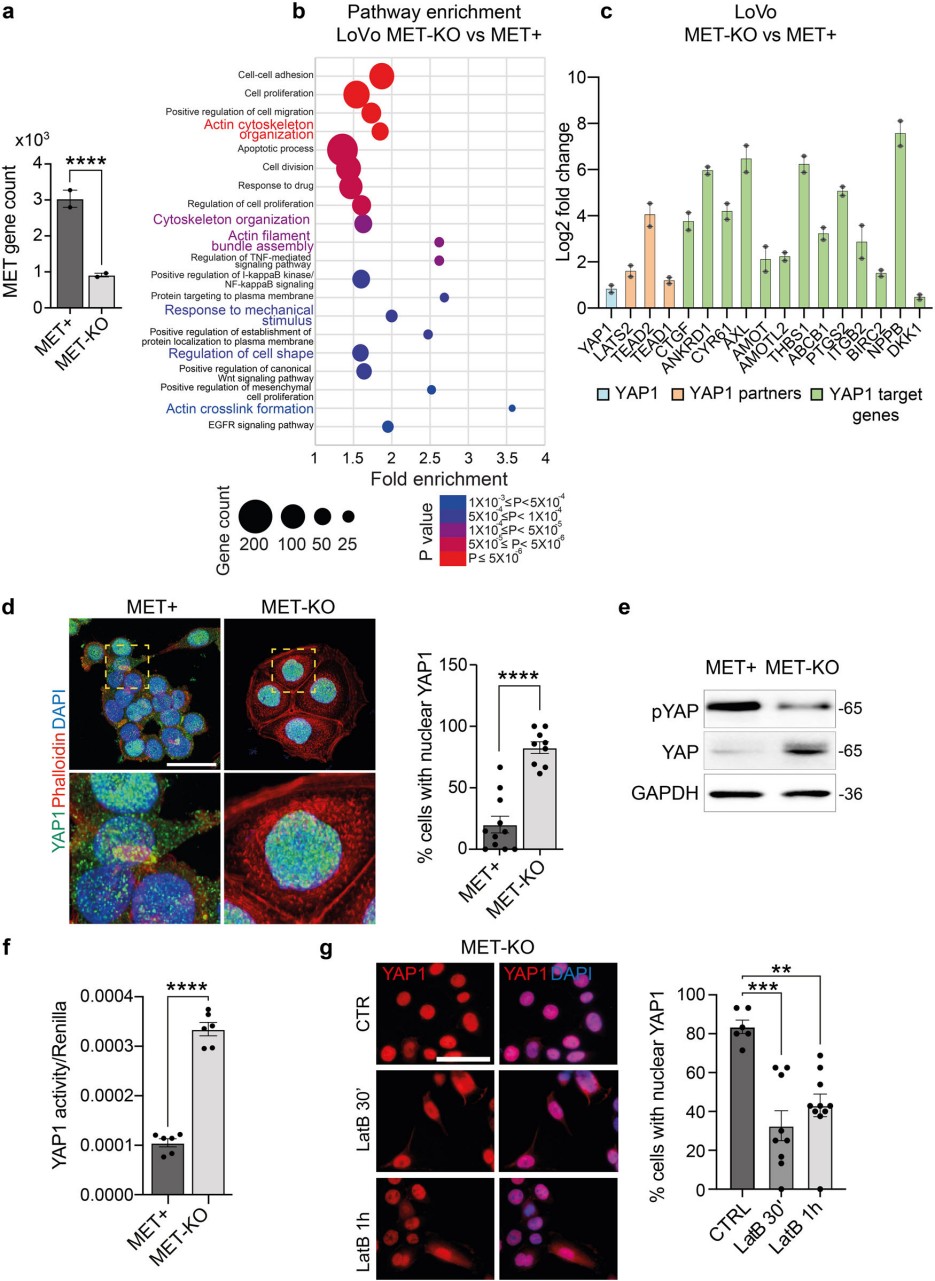
MET+ cells show reduced YAP1 nuclear localization and co-transcriptional activity. **a** *MET* (ENSG00000105976) gene count according to RNAseq data in MET+ and MET-KO cells. **b** Bubble chart of pathway enrichment analysis performed on RNAseq data showing the main pathways with differential expression between LoVo MET-KO vs MET+ cells. The size of the bubble correlates with gene count while the color represents statistical significance. Only pathways with *P* < 0.001 are shown. **c** Differential expression of known YAP1-related genes and YAP1-target genes in MET+ and MET-KO cells according to RNAseq data. Data are shown as Log2 fold change ± SDERR. **d** Evaluation of YAP1 subcellular localization in MET+ and MET-KO cells by means of YAP1 immunostaining (green). Nuclei were counterstained with DAPI (blue) and actin cytoskeleton was stained by Phalloidin (red). The quantification provides the % of cells showing a complete nuclear localization of YAP1, measured in at least 8 not overlapping fields per cell line. Data are shown as mean ± SEM. Statistic was calculated by T-test. A total of 300 cells was analyzed. Scale 'bar: 25 μm. **e** Western blot analysis of YAP1 protein levels and YAP1 Ser127-phosphorylation in MET+ and MET-KO cells. GAPDH was used as loading control. **f** Analysis of YAP1 activity in MET+ and MET-KO cells by luciferase assay with 8xGTIIC-luciferase reporter plasmid. Renilla luciferase was used as normalizing transfection control. Data are shown as mean ± SEM and statistic was calculated by T-test. **g** YAP1 (red) and DAPI (blue) staining in LoVo MET-KO cells treated with 80 nM Latrunculin B for 30’ and 1 h. The quantification was performed on at least 6 fields per condition and shows the % of cells with a complete nuclear localization of YAP1. Data are reported as mean ± SEM. Statistic was calculated by One-way ANOVA. A total of 300 cells were analyzed. Scale bar: 50 μm.

### MET activation controls YAP1 nuclear export and transcriptional activity

**Fig. 6:**
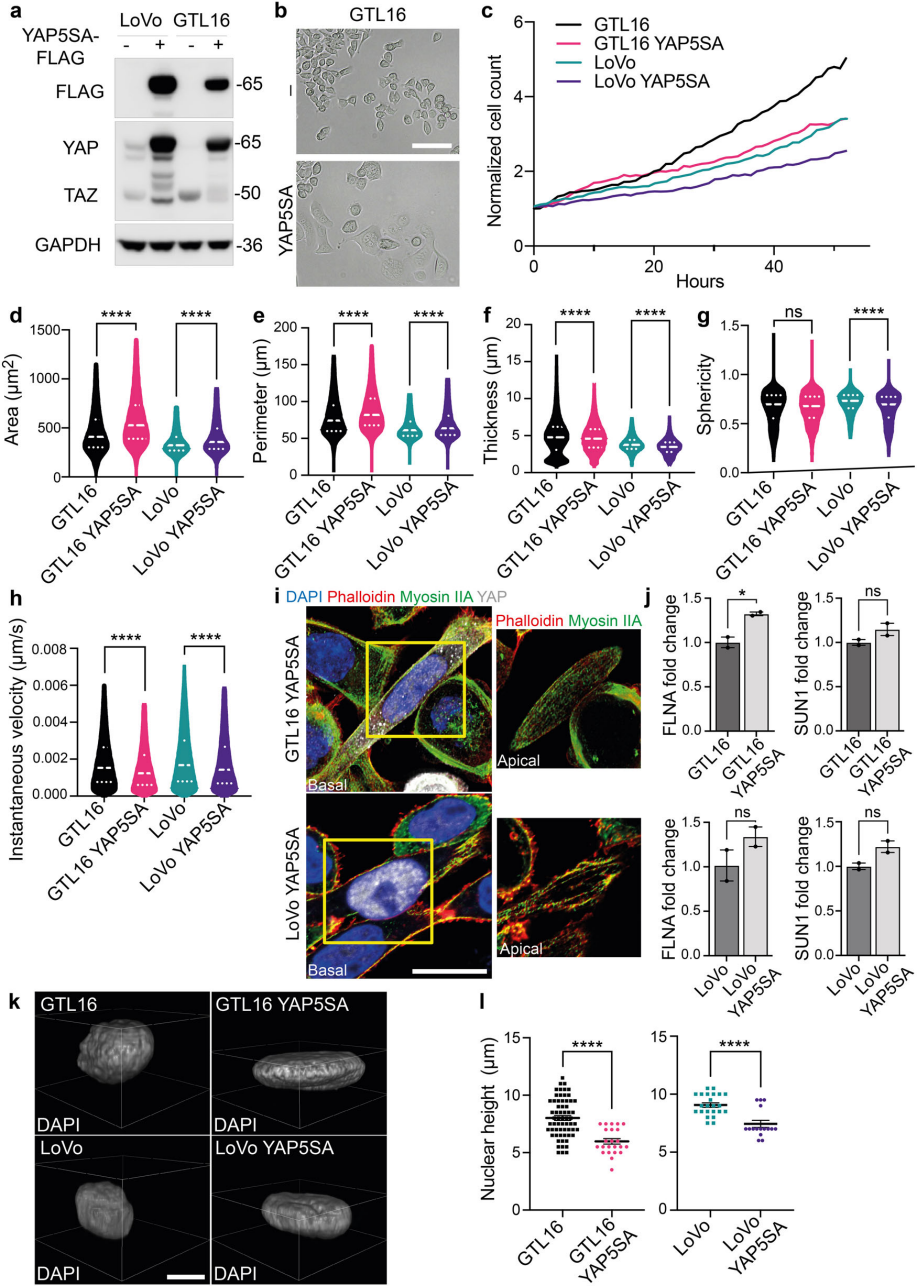
Mutated constitutively active YAP (YAP5SA) prompts actin cap formation and nuclear flattening. **a** Western blot analysis to validate YAP5SA insertion in LoVo and GTL16 cells. GAPDH was used as loading control. **b** Micrographs of WT and YAP5SA GTL16 cells. Scale bar: 50 μm. **c** Normalized cell count of LoVo and GTL16 cells infected with YAP5SA containing vector in a live-cell imaging experiment performed with Phasefocus Livecyte^TM^. Cells were monitored for more than 48 h and pictures were taken every hour. **d**–**h** Area, perimeter, thickness, sphericity and instantaneous velocity of LoVo and GTL16 cells WT and YAP5SA calculated from live-cell imaging in (**c**). Median and quartiles distribution are plotted. Outliers were identified and cleaned from results by means of ROUT method (Q = 1%). 5100 cells were analyzed. Statistic was calculated by T-test. **i** Confocal imaging of LoVo and GTL16 YAP5SA stained with DAPI (blue), Phalloidin (red), Myosin IIA (green) and YAP antibody (grey). A detail of the apical plane of section of cells is reported on the right. Scale bar: 10 μm. **j** *FLNA* and *SUN1* expression analysis by qPCR in GTL16 and LoVo YAP5SA expressing cells. *B2M* was used as housekeeping gene for normalization. T-test was used for statistical analysis. **k**, **l** Analysis of nuclear height in YAP5SA expressing cells. Representative images are reported in (**k**), while quantification is showed in (**l**) and was performed as described in Fig. 2D. 120 cells were analyzed. Statistic was calculated by T-test. Scale bar: 10 μm. The scale bar refers to the xy-plane.

Aiming to characterize the genetic profile driving these striking morphological changes, we performed RNA sequencing analysis in MET+ and KO cells. Interestingly, MET was among the most significantly downregulated genes in MET-KO cells, indicating an impaired stability of the mRNA induced by the CRISPR/Cas9 point mutation (Fig. 5a). The pathway enrichment analysis revealed an involvement of several signaling cascades, including those responsible in actin cytoskeleton organization, such as actin filament bundle assembly and actin crosslink formation, as well as regulation of the cell shape. Intriguingly, also the biological processes entangling cell response to mechanical stimuli appeared to be differentially expressed between the two models (Fig. 5b). Prompted by these results, we analyzed the involvement of YAP1, which transduces mechanical cues into gene expression programs^22^. Strikingly, a wide set of YAP1-regulated transcriptional targets, including the genes *CYR61*, *ANKRD1*, *THBS1*, *PTGS2*, *CTGF*, *NPPB* and *AXL*, appeared robustly upregulated in MET-KO cells compared to MET+ cells (Fig. 5c). Thus, we tested YAP1 activation by evaluation of subcellular localization. YAP1 was shown to localize primarily in the nucleus in MET-KO cells, while having a mixed diffuse cytosolic/nuclear localization in the MET+ cells, indicating a lower activation of the pathway due to cytoplasmic shuttling (Fig. 5d). Accordingly, YAP1 total form was decreased in protein lysates from aberrant MET+ compared to KO cells, compatibly with higher levels of YAP1 degradation following its relocation to the cytoplasm (Fig. 5e). Interestingly, the levels of YAP1 inhibitory phosphorylation on Ser127, resulting from LATS kinases cascade activation upon Hippo pathway engagement, were shown to be only slightly decreased in MET-KO model (Fig. 5e). In line, experiments with a luciferase reporter for YAP1 activity (8xGTIIC-Lux) showed a massive increment of YAP1 activation in basal conditions in KO cells compared to the MET+ cells (Fig. 5f). Following the strong inactivation of YAP1 observed in LoVo cells, we decided to evaluate YAP1 localization and protein levels also in an additional MET-dependent gastric carcinoma model, GTL16. GTL16 cells showed a massive protein expression and high phosphorylation levels of MET, while YAP1 was barely detectable (Supplementary Fig. 2d) most likely due to an increased trafficking to the cytoplasm (Supplementary Fig. 2e), where YAP1 degradation occurs. In addition, MET rescue in LoVo MET-KO cells by introduction of TPR-MET was sufficient per se to hamper YAP1 signaling by inducing YAP1 cytosolic relocation (Supplementary Fig. 4i). As a result, several YAP1 target genes were highly downregulated in rescued cells, mimicking YAP1 status before MET silencing (Supplementary Fig. 4j). Collectively these data suggest that MET hyperactivation is sufficient to induce YAP1 cytosolic localization, thus impairing its co-transcriptional activity. We further proved that upon MET ablation, cells experience strong dependency on YAP nuclear localization. Indeed, when forcing YAP to re-localize to the cytoplasm by changing the stiffness of the substrates and employing a soft ECM, namely matrigel or agar, MET-KO cells completely lost the capability to grow (Supplementary Fig. 6a–d, Supplementary videos 9, 10). Finally, impairment of the perinuclear actin cap by administration of low doses of LatB in KO cells, was sufficient to induce YAP1 cytoplasmic translocation (Fig. 5g). To sum up, we confirmed that MET ablation induces subcellular nuclear localization of YAP1, thus modulating its activity and the production of genes responsible for the perinuclear actin cap formation^23,24^. Next, an exogenous form of YAP harboring five serines-to-alanines mutations (5SA) that prevent the inhibition and degradation induced by phosphorylation, was inserted in LoVo and GTL16 cells and confirmed by western blot (Fig. 6a). Notably, YAP activation finely reproduced the MET silencing, being particularly evident in GTL16 cells, where marked differences in the cell phenotype are already visible with optical microscopy (Fig. 6b). The proliferation rate of both LoVo and GTL16 cells was found to be reduced by YAP5SA insertion, supporting the model that YAP could hold an anti-proliferative role in gastrointestinal cancer cells (Fig. 6c). Live-cell imaging assays revealed a significant increase in cell area (Fig. 6d), perimeter (Fig. 6e), but a decrease in thickness (Fig. 6f) and in the sphericity (Fig. 6g) in both GTL16 and LoVo cells. Moreover, cell velocity was found to be statistically decreased in both cell lines following the introduction of YAP5SA (Fig. 6h). To sum up, the constitutive activation of nuclear YAP blunted the main effects of oncogenic MET. Nonetheless, further investigations will be required to elucidate the mechanism by which MET/YAP regulates the preferential migratory pattern of these cells. Then we asked whether the introduction of constitutively active YAP1 was also sufficient to induce a correct alignment of the actin cap fibers, that are aberrant in LoVo and GTL16. The perinuclear actin cap was detected in both LoVo and GTL16 YAP5SA, as visualized through Phalloidin and Myosin IIA staining in the apical frame (Fig. 6i, details). In line, a mild although not statistic increase in *FLNA* and *SUN1* expression was detected in YAP5SA expressing cells (Fig. 6j). Due to the alignment of the actin cap and the resulting nuclear flattening, nuclear height was markedly reduced in YAP5SA-expressing cells (Fig. 6k, l). Altogether, YAP5SA introduction in MET+ hyperactivated cells was shown to reproduce the phenotype of MET-KO cells, allowing to restore the actin cap fibers aberrations induced by MET. These data suggest a causative role for YAP1 in the organization of the perinuclear actin cap, supporting the model that YAP inhibition could be a direct downstream effect of MET signaling activation in gastrointestinal cancer cells.

### MET activation in normal cells drives perinuclear actin rearrangement and YAP1 deactivation

**Fig. 7:**
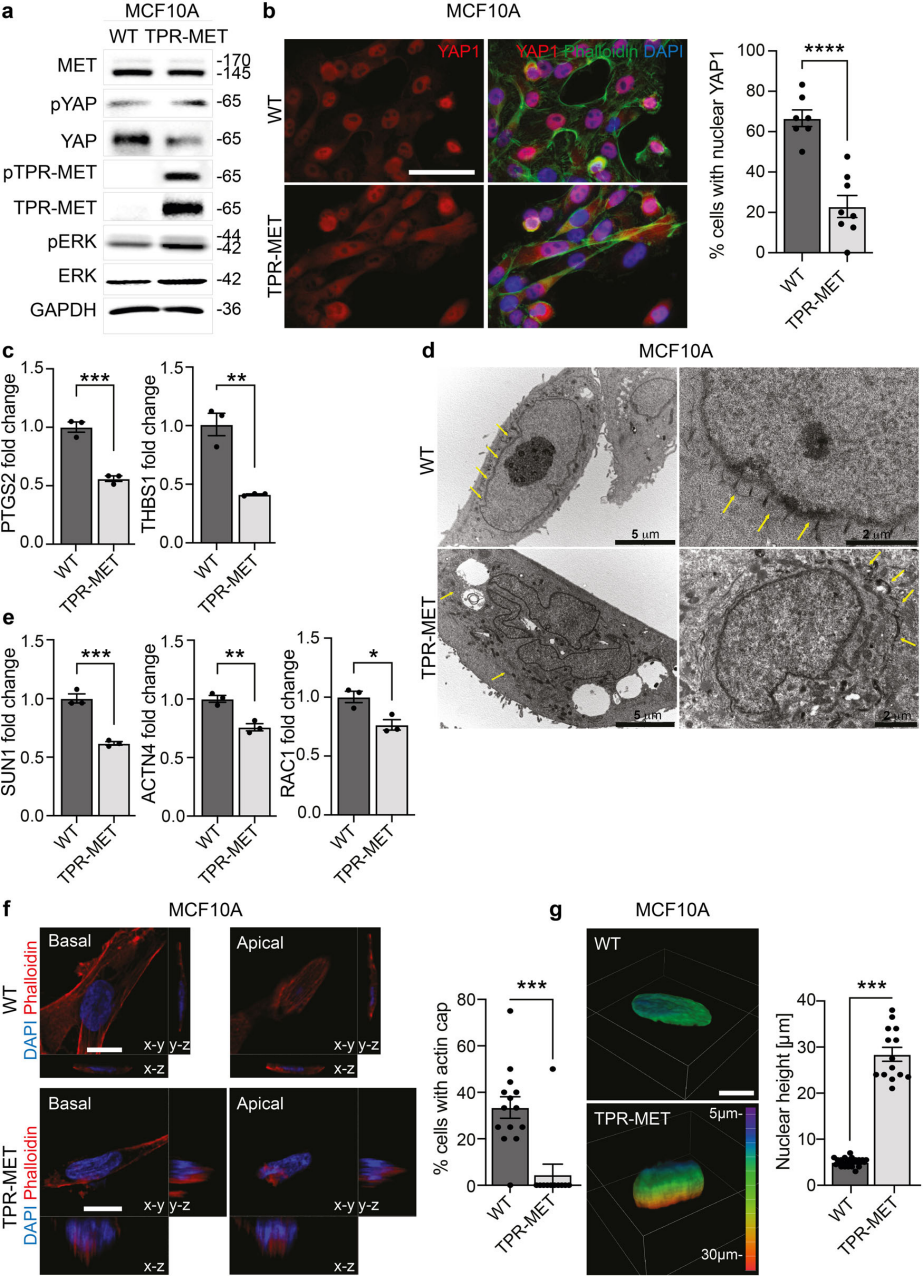
MET+ constitutive activation in normal cells induces YAP1 cytosolic translocation and actin cap disassembly. **a** Evaluation of YAP1 protein levels and phosphorylation on Ser127 in MCF10A WT and TPR-MET cells. The introduction of constitutively phosphorylated 65 kDa TPR-MET in MCF10A is confirmed together with downstream MAPK signaling activation. **b** Evaluation of YAP1 subcellular localization in MCF10A WT and TPR-MET cells. Cells were counterstained with Phalloidin (green) and DAPI (blue). The % of cells with complete nuclear YAP1 is reported in the quantification on the right. At least 7 independent fields were analyzed and the quantification relies on a total of 158 cells. Data are shown as mean ± SEM. Statistic was calculated by T-test. Scale bar: 50 μm. **c** Analysis of YAP1 target genes *PTGS2* and *THBS1* RNA expression by qPCR in MCF10A WT and TPR-MET. Data are shown as fold change in relation to MCF10A WT ± SD. Unpaired T-test was applied for statistical analysis. **d** Transmission electron microscopy of MCF10A WT and TPR-MET. Yellow arrows point at actin cap filaments. Individual scale bar is indicated for each image. **e** Determination of and actin cap-related genes *SUN1*, *ACTN4* and *RAC1* expression by qPCR in MCF10A WT and TPR-MET. Data are reported as fold change in relation to MCF10A WT ± SD. Statistic was calculated by T-test. **f** Analysis of actin cap presence in the apical xy-plane of MCF10A WT and TPR-MET by means of Phalloidin (red) and DAPI (blue) staining. yz and xz Z-stacks are also reported. The quantification shows the % of cells with properly assembled actin cap, as measured in at least 11 fields per cell line. For MCF10A TPR-MET, only cells highly positive to pMET cytosolic staining were analyzed. A total of 100 cells were analyzed. Statistic was calculated by T-test. Scale bar: 10 μm. **g** Assessment of nuclear height in MCF10A WT and TPR-MET from experiment in (**d**). Quantification was performed by analyzing Z-stacks images of 40 cells. Representative 3D rendering images are provided, and Z-depth coding is reported as a scale of colors. Statistic was calculated by T-test. Scale bar: 10 μm. The scale bar refers to the xy-plane.

Finally, we engineered the *quasi*-normal epithelial cell line MCF10A, that displays a regular alignment of the apical stress fibers (Supplementary Fig. 7a), with the constitutively active fusion protein of MET, TPR-MET^19^. TPR-MET guarantees that MET signaling is constitutively activated, even in absence of HGF ligand administration and the correct introduction in MCF10A cells was validated by immunofluorescence (Supplementary Fig. 7b) and Western Blot (Fig. 7a). Interestingly a boosted downstream activation of the MAPK was also detected (Fig. 7a). In addition, the stimulation of MET axis caused by TPR-MET insertion was per se sufficient to reduce YAP1 total protein levels and to slightly enhance YAP1 phosphorylation on Ser127 (Fig. 7a). Consistently, in cells stably expressing TPR-MET, immunofluorescence analysis revealed a cytosolic shuttling of YAP1 in contrast with the nuclear localization detected in MCF10A reference cells (Fig. 7b). Notably, also YAP1 activity appeared halted in TPR-MET cells, which display a downregulation of YAP1 target genes *PTGS2* and *THBS1* (Fig. 7c). Moreover, by activating MET axis with HGF in MCF10A cells, we recorded an increased YAP1 degradation along with a decreased nuclear subcellular localization (Supplementary Fig. 7c, d). In line, MET interception by the kinase inhibitor capmatinib (CAP) in MET+ cells induced an increase in YAP1 protein abundance, although the role of MET kinase activity in the switch from the actin cap to the actin patches remain to be addressed (Supplementary Fig. 7e). Next, Transmission Electron Microscopy (TEM) was used to visualize the cytoskeleton components in MET+ constitutively activated MCF10A in comparison with naïve cells. Interestingly, MCF10A presented a neat pattern of repeated perinuclear sites, with an ordered spatial organization of about 400 nm apart, probably representing the indentation marks of the actin filament fibers covering the nuclear envelope and forming the actin cap (Fig. 7d). The introduction of aberrant MET altered this organization, the neat pattern of indentation marks disappeared by leaving concentrical aggregates far away from the nuclear envelope along with an irregular nuclear shape **(**Fig. 7d). Moreover, the actin cap-related genes *SUN1*, *RAC1* and *ACTN4* were found to be markedly decreased in MCF10A-TPR-MET compared to parental cells (Fig. 7e). Finally, we further proved that loss of perinuclear apical actin stress fibers in MCF10A-TPR-MET (Fig. 7f) associated with a pronounced nuclear height, probably resulting from the deficit of actin cap-mediated vertical confinement (Fig. 7g).

## Discussion

Understanding the physical properties of cells become increasingly important when analyzing the metastatic dissemination of cancer cells from primary tumors to distant sites. During metastatic spread, cells manage to pass through several forms of physical confinement, particularly during the intra- and extravasation processes, producing a high mechanical stress. In this context, an impaired nuclear architecture integrity correlates with genomic instability and cancer cell aggressiveness^25^. Moreover, nuclear and cell shape modifications modulate an intricate pattern of mechanically induced transcriptional programs and downstream signaling pathways. Several elements are known to contribute to the regulation of the nuclear architecture, first and foremost the forces provided by cytoskeletal tensions, acting through physical connections between the apical actin filaments covering the nucleus, the so-called perinuclear actin cap, and the nuclear envelope^4^. So far, the exact biological processes through which the actin cap is regulated are still unknown. In this work, we propose a role for RTKs in the proper assembly of the perinuclear actin cap and the correct organization of the nuclear structure. By employing a cellular model characterized by a constitutive activation of MET receptor, we recorded a previously undescribed highly dynamic aberrant perinuclear actin structures originating by the dismantling of the actin cap-derived stress fibers. These actin patches likely originate from the invagination of the cell membrane due to the coalescing of actin filaments and are mainly constituted by apical microvilli. These patches were found to be associated with an increased nuclear height, as well as reduced focal adhesion stabilization and random meandering migration. By means of MET ablation, we were able to demonstrate that even in aggressive cancer cells, it is possible to restore a properly aligned actin cap, undistinguishable from the one described in cultured fibroblasts and normal epithelial cells. According to these findings, we anticipate that the mechanical stress shear encountered by primary tumor cells during dissemination could exploit the MET-driven actin aberrations which fail to protect the nucleus from DNA damage and prompting hypermutability.

Mechanistically, we found an unexpected association between MET axis and YAP1 inhibition, further confirmed by the introduction of a constitutively active MET (TPR-MET) in a normal epithelial model. Despite YAP1 is generally considered as an oncogenic factor, its role in cancer progression is still controversial, with a growing body of evidence pointing to YAP1 as a tumor suppressor^26–29^. Notably, YAP1 has been found to suppress tumor growth in vitro and in vivo by reprogramming intestinal stem cells^29^. In support to the proposed model depicting YAP1 inactivation as a mediator of MET-induced perinuclear actin dismantlement, recent findings shed light on the regulation of this structure. In detail, YAP1 was shown to play a proactive role in the arrangement of the perinuclear actin cap, by inducing the transcription of ACTR2. This gene is responsible for encoding the master regulator of actin polymerization, acting as a crucial component of the ARP2/3 complex^30^. Although we did not observe an involvement of ACTR2 in the cell models employed, our study supports a causative role of MET-mediated YAP1 cytosolic shuttling in the disruption of the perinuclear actin cap. In line with our findings, a comprehensive study in hepatocytes derived from transgenic mice reported that the inducible constitutive mutated YAP1 (YAPS127A) was able to control the production of the actin cap structural protein FLNA (filamin A)^31^. In our hands, the forced introduction of nuclear YAP rescued the actin cap aberrations induced by oncogenic MET, thus counteracting its function. Consistently, MET-KO cells with activated YAP1 show high level of FLNA and compressed nuclei. Of note, FLNA attaches actin fibers to each other, thereby controlling cell rigidity and it also anchors transmembrane proteins, such as integrins^32^. The tensions provided by the proper alignment and anchoring of the actin cap may further drive YAP1 nuclear shuttling, which in turn activates the production of perinuclear actin cap genes, with implications in persistent cell motility and sustained YAP1 activity as previously reported^33,34^.

The ultimate biological effect induced by MET-driven perinuclear actin cap dismantling is the inhibition of persistent migration, in favor of an amoeboid-like motility characterized by scattered and continuous changes of direction. Of note, in line with our data, YAP1 was previously shown to be involved in the regulation of persistent cell motility^34^. In turn, we suggest that the amoeboid migratory pattern that derives by MET hyperactivation and/or YAP1 inhibition could promote cell invasion by facilitating cells squeezing and migration in confined environments, being also supported by proliferative and pro-survival signaling as reviewed here^35^.

All together, these data suggest a causative role for YAP1 in the organization of the perinuclear actin cap, with consequent polarization of cells during persistent movements^8^. Our model implies that YAP inhibition could be a downstream effect of MET signaling in gastrointestinal cancer cells.

How exactly the actin cap aberrations support metastasis and cancer progression in patients remains to be understood. Probably, the nuclear envelope exerts a protective role by preventing nuclear fragility and contrasting aging. YAP1 activation was shown to be the mediator of the mechanical pliability of the nuclear envelope. Finally, by ultrastructural imaging, we captured the perinuclear anchoring marks probably representing the attachment sites of the individual cap fibers covering the nuclear lamina. Their parallel orientation to the leading edge, suggests that these actin-rich spots may represent the anchoring site of previously described transverse arcs harboring dorsal fibers and multiple actin fibers, detectable in the cellular midsection and close to the nucleus^36^. We recorded a perinuclear localization and an impressively regular pattern of these anchoring marks, with an interspace of about 400 nm apart, supporting the model that these stretched actin fibers transmit mechanical stress to the nuclear lamina. Notably, aberrant MET directly interferes with the alignment of these actin filaments by disassembling the indentation marks and decreasing the amount of four structural components of the perinuclear actin cap. Consequently, MET by impairing the nucleus associated actin cap through the relocation of YAP1 in the cytosol, is able to control nuclear integrity and morphology (size and shape), the type of nuclear movements (rotation versus translocation), and the dominant mode of cell migration (persistent versus non-persistent).

## Methods

### Cell culture

LoVo cells were grown in Dulbecco’s Minimal Essential Medium (DMEM) high-glucose supplemented with 10% fetal bovine serum (FBS) and 1% penicillin-streptomycin. GTL16 cells were kindly provided by Prof. Silvia Giordano and grown in Roswell Park Memorial Institute (RPMI) 1640 medium, supplemented as well with 10% FBS and 1% penicillin-streptomycin. MCF10A cells were kindly provided by Prof. Yosef Yarden and grown in DMEM/F12 supplemented with 5% Horse Serum, Insulin (Sigma-Aldrich, St.Louise, Missouri, USA), Hydrocortisone (Sigma-Aldrich, St.Louise, Missouri, USA) and EGF 10 ng/mL. LoVo cells were validated with the external service Eurofins Medigenomix, Ebersberg. Cells were cultured in a humidified incubator at 37 °C with 5% CO_2_. All cell lines were tested for *Mycoplasma* contamination by PCR.

### Immunoblotting

For immunoblotting, cells were lysed using RIPA buffer supplemented with 1 mM Na_3_VO_4_ (Santa-Cruz Biotechnology, Dallas, USA) and a protease inhibitor cocktail (P8340, Sigma-Aldrich, St.Louise, Missouri, USA). DC Protein Assay (Bio-Rad, Hercules, USA) was employed to assess protein concentration, using bovine serum albumin as the standard. Protein samples were separated by SDS-PAGE and then transferred to nitrocellulose membranes (Amersham™ Protran™ Premium, GE Healthcare, USA). Blocking was performed by incubating membranes for 1 h with 5% BSA in TBS-T (0.1% Tween-20) and incubated O/N at 4 °C with primary antibodies. The full list of the antibodies used is provided in Supplementary table 1. Eventually, membranes were incubated for 1 h with horseradish peroxidase-coniugated secondary antibodies (mouse or rabbit) and protein bands were detected by chemiluminescence (Amersham™ ECL™ Detection Reagents).

### CRISPR-Cas9 gene silencing

For cells knock-out, the CRISPR/Cas9 system derived from *Streptococcus pyogenes* was used, following the protocol developed by Ran and colleagues^15^. Oligo’s sequences used for PCR-based sgRNA construction are reported in Supplementary Table 2. PCR for sgRNA-encoding DNA amplification was carried out with high-performance enzyme from KAPA HiFi HotStart PCR Kit (Sigma Aldrich, St.Louise, Missouri, USA) and a purification step was performed using GenElute^TM^ PCR Clean-Up Kit (Sigma Aldrich, St.Louise, Missouri, USA). LoVo cells were then co-transfected with pSpCas9-puro plasmid, gently provided by Roychoudhuri Laboratory, Babraham Institute, Cambridge, pGFP (for transfection control) and MET sgRNA-encoding DNA. Lipofectamine 2000 (Invitrogen Co., Waltham, Massachusetts, USA) was employed to allow DNA entry into cells. Antibiotic selection was performed with 2,5 µg/mL Puromycin. Single clones were manually picked and grown separately. KO clones were selected by Western Blot.

### Luciferase reporter assay

Luciferase reporter assay was employed to evaluate YAP1 activity in cultured cells. 10.000 cells were seeded in 96-wells plate and, the day after, transfected with 200 ng of 8xGTIIC-luciferase reporter^23^, kindly provided by Prof. Michelangelo Cordenonsi (University of Padua), by means of Lipofectamine 2000 (Invitrogen Co., Waltham, Massachusetts, USA) according to manufacturer’s instructions. 24 h after transfection, cells were incubated for 1 h with Beetle Luciferin 100 μg/mL (Promega, Madison, Wisconsin, USA) and signal outputs were detected with Victor^TM^ Multilabel Plate Reader (PerkinElmer Inc.). Renilla expressing plasmid was co-transfected in cells and used to normalize luciferin signal over transfection efficiency.

### Retroviral and lentiviral transduction

pTK93_Lifeact-mCherry plasmid was obtained from Prof. Yosef Yarden Laboratory, as used here^37^ while pBABE-puro TPR-Met was purchased from Addgene. Hygromycin-selected Phoenix cells were used as packaging cells to produce retroviral particles. Retroviral vectors were transfected in packaging cells using calcium phosphate transfection protocol, and chloroquine 25 μM was employed to increase transfection efficiency. Retroviral supernatants were withdrawn, filtered, and used to infect target cells previously seeded in full media. Polybrene (Sigma Aldrich, St.Louise, Missouri, USA) 5 μg/mL was employed to increase infection yields.

Flag-hYAP-5SA mutant cDNA was subcloned in the lentiviral empty vector CSII-CMV-MCS-IRES-bsd (a gift from H. Miyoshi). For lentiviral particles preparation, HEK293T cells were seeded at 40% confluency in 10cm-dishes and co-transfected with packaging plasmids pMD2-VSVG (2.5 ug) and pPAX2 (7.5 ug), together with lentiviral vectors (10 ug). Transfection was performed with TransIT-LT1 reagent (Mirus Bio LLC, Madison, Wisconsin, USA) following manufacturer’s instructions. Lentiviral supernatants were withdrawn, filtered, and used to infect target cells previously seeded in full media in a 1:4 ratio of lentiviral supernatant:full culture medium.

### Immunofluorescence

For standard immunofluorescence, 30/40.000 cells were seeded in full medium onto glass coverslips and incubated according to the experiment. At end point, cells were fixed in 4% PFA, permeabilized with 0.2% Triton and then incubated for 1 h in BSA 1%. Incubation with primary antibodies was carried out overnight in humidified chamber. The day after, coverslips were incubated for 1 h with fluorescence-conjugated secondary antibodies, and then for 30’ with DAPI (Sigma Aldrich, St.Louise, Missouri, USA) and FITC-/TRITC-conjugated Phalloidin (Sigma Aldrich, St.Louise, Missouri, USA). Primary and secondary antibodies used for immunofluorescence are available in Supplementary table 1. Images were taken using both the Olympus BH-2 CCD microscope and a Nikon A1-R confocal laser scanning microscope. Volume view with 3D rendering was carried out using the NIS Elements Advanced Research software (Nikon, Shinagawa, Japan). Super resolution imaging was carried out with CrestOptics X-Light V3 Spinning Disk equipped with super resolution module CrestOptics DeepSIM (CrestOptics, Rome, Italy).

### Transmission Electron Microscopy (TEM)

For the ultrastructural analysis of MCF10A and MCF10A TPR-MET, 400.000 cells were seeded in a 6-wells plate and incubated for 36 h. Then, cells were directly fixed in 6-well plate with 2.5% buffered glutaraldehyde for 20’ at room temperature, scraped from the culture well, harvested and centrifuged before the storage at 4 °C O/N in the same fixative. Then, pellets were rinsed in phosphate buffer, post-fixed in 1% buffered osmium tetroxide for 1 h at RT, subjected to dehydration by ethanol at increasing concentrations and embedded in Araldite resin. After sectioning, 80 nm ultrathin sections were recovered on formvar coated grids, counterstained with uranyl acetate and lead citrate, and examined in a Philips CM100 (FEI Company, ThermoFisher, Waltham, MA, USA) Transmission Electron Microscope obtaining digital images using an Olympus camera.

### Phasefocus livecyte™

For Phasefocus Livecyte™ imaging, 2–3000 cells were seeded in full medium in 96-wells plates and incubated overnight to allow cells’ adhesion before starting Livecyte™ monitoring. Images of cells were taken at 10X magnification every 4 h for a total of 2 days. Proliferation, random motility, and cell morphology parameters were computed by Phasefocus Livecyte™ software.

### Confocal live-cell imaging

LoVo cells were seeded onto poly-L-lysine-coated (0.1 mg/ml) coated glass-bottom dishes (MatTek, USA), mounted on a dedicated stage incubator (OkoLab, Italy). The experiments were performed on a Nikon A1-R confocal laser scanning microscope (Nikon, Tokyo, Japan) equipped with a perfect focus system, a PlanApo VC 60× NA 1.4 oil immersion objective lens and 561 nm laser line. The confocal images were acquired with resonant scanner at optical resolution of 210 nm/pixel, stored at 12-bit with 4096 different grey levels, pinhole diameter set to 2 Airy unit, and z-step size to 1 μm. Fluorescent detection was merged to brightfield channel to identify cell compartments. For time-lapse acquisition the images were captured every 10 min and processed by using NIS-Elements Advanced Research software (Nikon, Tokyo, Japan) ver. 5.20.

### Optical diffraction tomography

Lovo LifeAct cells MET+ and MET-KO were seeded on a glass-bottom Petri dish for the Optical Diffraction Tomography (ODT) imaging (HT-1H, Tomocube Inc., Republic of Korea). Before performing ODT imaging, cells were washed with phosphate-buffered saline (PBS) and stained with Phalloidin/DAPI for 30 min at room temperature. After washing, fresh medium DMEM + 10% FBS was reintroduced in the Petri dish. An in-situ incubator attached to the imaging setup was used for keeping the cells alive during the experiment. Cells were observed using a 60× NA 1.2 water immersion objective lens. The hologram acquisition was captured as individual holographic images of a sample that had been illuminated from different angles by scanning object illuminating beam while the camera and the sample are stationary. An interferometric setup with a digital micromirror device was used^38^. The refraction index (RI) distribution of the samples was reconstructed using an ODT algorithm based on the Fourier diffraction theorem^39^.

For time-lapse acquisition the images were captured every 10 min and the planes passing through the center of the cells were selected, aligned and crop to create a frame animation. The 3D distribution of the refractive index and fluorescence of live cells and their 2D maximum intensity projections were generated, visualized, and analyzed with TomoStudio software (Tomocube Inc, Daejeon, Korea). For the morphological analysis of the cells, TomoStudio^TM^ (version 2.7) software was used.

### RT-qPCR and RNA sequencing

For both qPCR and RNA sequencing, total RNA was extracted from cells with QIAzol® Lysis Reagent (QIAGEN, Venlo, Netherlands) following manufacturer’s instructions. qRT-PCR analysis was performed as ΔCq (Quantification Cycle). ΔCq were normalized on the expression of β_2_-microglobulin (B2M) housekeeping gene. A list of the primers used is available as Supplementary Table 3.

RNA sequencing was performed by Lexogen GmbH. In detail, RNA libraries were prepared using QuantSeq 3’ mRNA-Seq Library Prep Kit (FWD) following manufacturer’s protocols.

### siRNA transfection

SUN1/2 and SYNE1/2 siRNA mix were validated in^30^. The full sequence is reported as Supplementary table 4. siRNAs were transfected in target cells by Lipofectamine 2000 (Invitrogen Co., Waltham, Massachusetts, USA) transfection according to manufacturer’s instructions. 24 h after transfection, cells were detached and re-seeded according to the experiment.

### Bioinformatical analyses

Colon cancer gene expression datasets were downloaded from GEO^18^. GEO-derived samples had gene expression based on gene arrays. Pearson and Spearman correlation coefficients were computed to correlate the gene expression values of different genes.

### Statistics and reproducibility

The statistical analyses were performed by using Prism version 6 (GraphPad Software, Inc). T-test, one-way and two-way ANOVA were used to assess significance of the tests. Details about the applied statistical test are reported in the figure legends for each assay. *P-value < 0.05; **P-value < 0.01; ***P-value < 0.001; ****P-value < 0.0001.

### Inclusion & ethics statement

All co-authors of this publication have met the criteria for authorship required by Nature Portfolio journals as reported in the contributions section and as agreed among collaborators beforehand. This research does not result in stigmatization, discrimination, incrimination, or personal risk to participants.

### Reporting summary

Further information on research design is available in the Nature Portfolio Reporting Summary linked to this article.

### Supplementary information

Supplementary information

Description of Additional Supplementary Files

Supplementary Data 1

Supplementary Video 1

Supplementary Video 2

Supplementary Video 3

Supplementary Video 4

Supplementary Video 5

Supplementary Video 6

Supplementary Video 7

Supplementary Video 8

Supplementary Video 9

Supplementary Video 10

Reporting Summary

## Data Availability

The RNAseq data discussed in this publication have been deposited in NCBI’s Gene Expression Omnibus^40^ and are accessible through GEO Series accession number GSE212835. All other source data supporting the findings of this study are available within the paper and its Supplementary Information. Uncropped blots are provided as Supplementary Fig. 8. Source data underlying the graphs and charts presented in the main figures are provided as Supplementary Data 1.
