# Supplementary material for: Aberrant MET activation impairs perinuclear actin cap organization with YAP1 cytosolic relocation
Source: Commun Biol. 2023 Oct 14;6:1044. doi: 10.1038/s42003-023-05411-y (PMC10576810; doi:10.1038/s42003-023-05411-y)
Supplement: Supplementary file 1 — Supplementary information [file 42003_2023_5411_MOESM1_ESM.pdf]

Supplementary information

ANTIBODY	SOURCE	CATALOG CODE
Phospho-AKT (Ser473) (D9E) XP Rabbit monoclonal antibody	Cell Signalling Technology	#4060
AKT Rabbit polyclonal antibody	Cell Signalling Technology	#9272
MAP Kinase activated (diphosphorylated ERK 1/2) Mouse monoclonal antibody	Sigma-Aldrich	M8159
ERK 2 (D-2) Mouse monoclonal antibody	Santa Cruz Biotechnology	sc-1647
Phospho-Met (Tyr1234/1235) (D26) XP ® Rabbit mAb	Cell Signalling Technology	#3077
Met (D1C2) XP ® Rabbit mAb	Cell Signalling Technology	#8198
Vinculin (7F9) mouse monoclonal IgG	Santa Cruz Biotechnology	sc-73614
Lamin A/C (E-1) Mouse monoclonal antibody	Santa Cruz Biotechnology	sc-376248
Anti-Myosin IIA, non-muscle antibody produced in rabbit	Sigma-Aldrich	M8064
YAP (63.7) mouse monoclonal IgG2a	Santa Cruz Biotechnology	sc-101199
Phospho-YAP (Ser127) Rabbit Ab	Cell Signalling Technology	#4911
Anti-GAPDH antibody produced in rabbit	Sigma-Aldrich	G9545
β-Actin (C4) Mouse monoclonal antibody	Santa Cruz Biotechnology	sc-47778
Anti-Mouse/Anti-Rabbit EnVision+ System- HRP Labelled Polymer	Dako	K4001/K4003
Alexa Fluor® 488 AffiniPure Goat Anti-Mouse IgG (H+L)	Jackson ImmunoResearch	115-545-003
Goat anti-Mouse IgG Secondary Antibody, Alexa Fluor 488	ThermoFisher Scientific	R37120
Alexa Fluor® 488 AffiniPure Goat Anti- Rabbit IgG (H+L)	Jackson ImmunoResearch	111-545-144
Goat Anti-Mouse IgG H&L (Alexa Fluor® 647)	Abcam	ab150115
Goat Anti-Rabbit IgG H&L (Alexa Fluor® 647)	Abcam	ab150079
HRP-conjugated anti-FLAG (Clone M2)	Sigma-Aldrich	A8592

Supplementary table 1 – List of primary and secondary antibodies used.

PRIMERS	5’⇨3’ SEQUENCE	AMPLIFIED PRODUCT LENGTH (bp)
U6-Fwd	GAGGGCCTATTTCCCATGATTCC	370
MET U6-Rev	AAAAAAAGCACCGACTCGGTGCCACTTTTTCAAGT TGATAACGGACTAGCCTTATTTTAACTTGCTATTTC TAGCTCTAAAAcGCGACATGTCTTTCCCCACACCGG TGTTTGCTCCTTTCCACAAG	

Supplementary table 2 – Oligos for PCR-based sgRNA construction

PRIMERS	5’⇨3’ SEQUENCE	SOURCE
B2M-FW	TGCCTGCCGTGTGAACCATGT	Sigma-Aldrich
B2M-RW	TGCGGCATCTTCAAACCTCCATGA	Sigma-Aldrich
PTGS2-FW	AAGCAGGCTAATACTGATAGG	Sigma-Aldrich
PTGS2-RW	TGTTGAAAAGTAGTTCTGGG	Sigma-Aldrich
THBS1-FW	GTGACTGAAGAGAACAAAGAG	Sigma-Aldrich
THBS1-RW	CAGCTATCAACAGTCCATTC	Sigma-Aldrich
SUN1-FW	GTGTTTCTTCTTACCAGGTG	Sigma-Aldrich
SUN1-RW	TAGTAAAAGGAAGAGTGGGATG	Sigma-Aldrich
ACTN4-FW	AGTATGACAAGCTGAGGAAG	Sigma-Aldrich
ACTN4-RW	CTGAAAAGGCATGGTAGAAG	Sigma-Aldrich
RAC1-FW	TTGGTGCTGTAAAATACCTG	Sigma-Aldrich
RAC1-RW	GGCATTTTCTCTTCCTCTTC	Sigma-Aldrich
SYNE1-FW	ACCAGGACATTGCATATTAC	Sigma-Aldrich
SYNE1-RW	TTTAAGGTTTCCCAAGAACG	Sigma-Aldrich
SYNE2-FW	GAGAAGATAGAAGAAGCACTC	Sigma-Aldrich
SYNE2-RW	TCTTATAGGTTTTCTGCTGC	Sigma-Aldrich
SUN2-FW	AGCCTTCAGATTCTCTTCAG	Sigma-Aldrich
SUN2-RW	ATTTCCTCTTTCAAGGTCCTG	Sigma-Aldrich
NPPB-FW	ATTAAGAGGAAGTCCTGGC	Sigma-Aldrich
NPPB-RW	AAATGAGTCACTTCAAAGGC	Sigma-Aldrich
CTGF-FW	TTAAGAAGGGCAAAAAGTGC	Sigma-Aldrich
CTGF-RW	CATACTCCACAGAATTTAGCTC	Sigma-Aldrich
CYR61-FW	TTGATTGCAGTTGGAAAAGG	Sigma-Aldrich
CYR61-RW	GCCTTGTAAAGGGTTGTATAG	Sigma-Aldrich
AXL-FW	CATGAAACATGGAGACCTAC	Sigma-Aldrich
AXL-RW	ATCTCTTGGTACTCAGATACTC	Sigma-Aldrich
ANKRD1-FW	TGAGTATAAACGGACAGCTC	Sigma-Aldrich
ANKRD1-RW	TATCACGGAATTCGATCTGG	Sigma-Aldrich

Supplementary table 3 – Primers for RT-qPCR

siRNA name	siRNA target sequence
siSYNE1_2	CTGCATAGTACCGAAACCCAA
siSYNE1_5	CAGGAGCTTCAGAGAGACATA
siSYNE1_7	ACCATCGAGCTCCAGATCAAA
siSYNE1_8	CTGGAGTGGGATCACGACTAT
siSYNE2_2	CAGAACTAGTTGATTAGTTTA
siSYNE2_4	CCCGAGCATCACTACAAGCAA
siSYNE2_5	CTGGTAGAACGTCAACCTCAA
siSYNE2_6	AAGGCTCATGTCACCGATCCA
siSYNE1/2	siSYNE1_2 + siSYNE1_5 + siSYNE1_7 + siSYNE1_8 + siSYNE2_2 + siSYNE2_4 + siSYNE2_5 + siSYNE2_6
siSUN1_1	CAGCAGCGCTGTCTCCCTGAA
siSUN1_4	CAGCGCAGAAGCACAAACAAA
siSUN1_5	ACCGAGCGGCCAGAACAACAA
siSUN1_6	CTGCAGGATGCTGTGACTCGA
siSUN2_6	CCGCATCGGGCTGGCAGACTA
siSUN2_7	CACCCGATGTTCTGAGACCTA
siSUN2_8	CCCGAAGAAGCCAGCGCCTCA
siSUN2_9	CAGCGTGTTATGTCCCGGGTA
siSUN1/2	siSUN1_1 + siSUN1_4 + siSUN1_5 + siSUN1_6 + siSUN2_6 + siSUN2_7 + siSUN2_8 + siSUN2_9

Supplementary table 4 – siRNA sequences

Supplementary figures

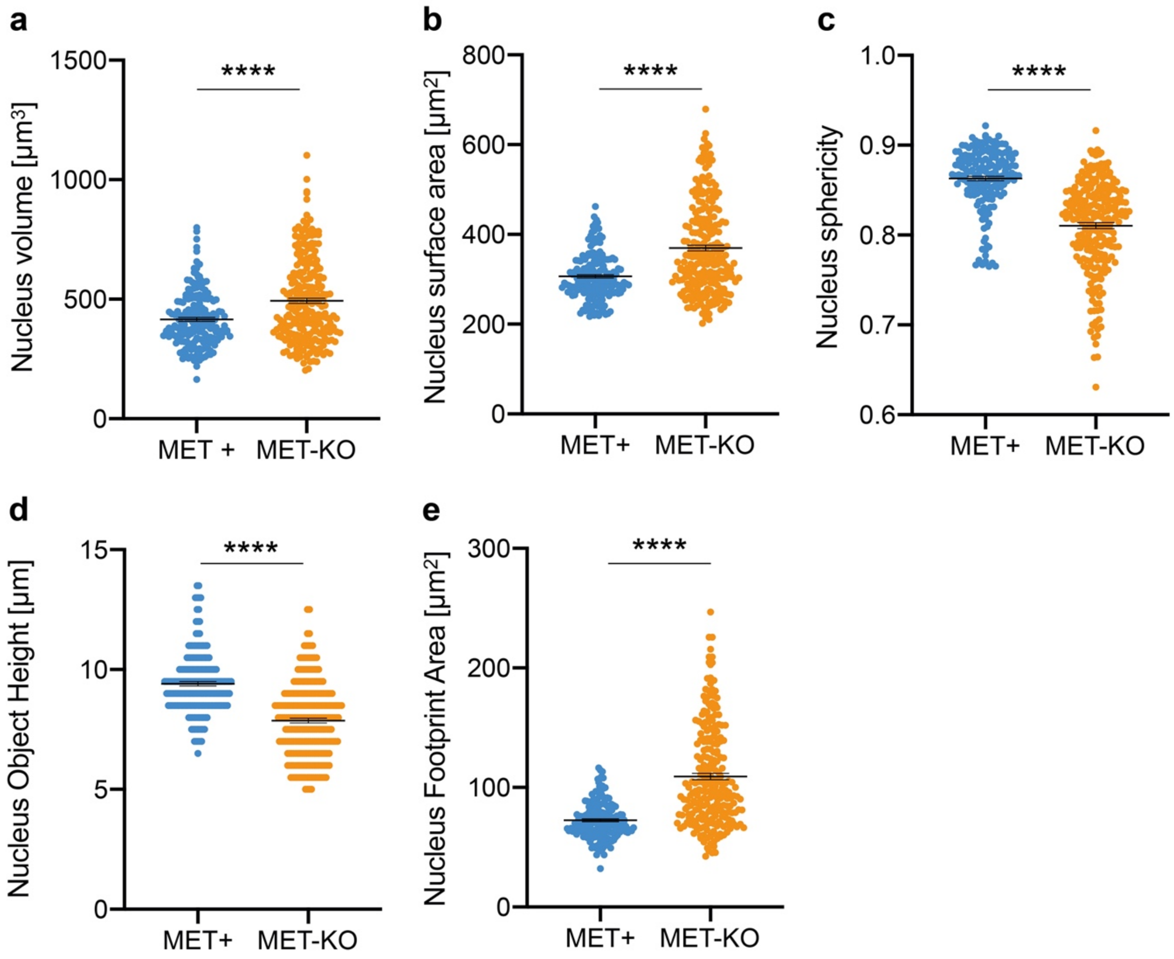

Supp. Figure 1: Nuclear morphology is deeply affected by MET silencing in LoVo cells. a, b, c, d, e) Fixed LoVo MET+ and MET-KO cells stained with DAPI and Lamin A/C were analyzed through Operetta CLS High-content screening system to measure several morphometric properties of the nuclei, namely nucleus volume (a), nucleus surface area (b), nucleus sphericity (c), nucleus object height (d) and nucleus footprint area (e). 189 and 247 cells for MET+ and MET-KO respectively were analyzed. Data are shown as mean ± SEM. Statistic was calculated by T-test.

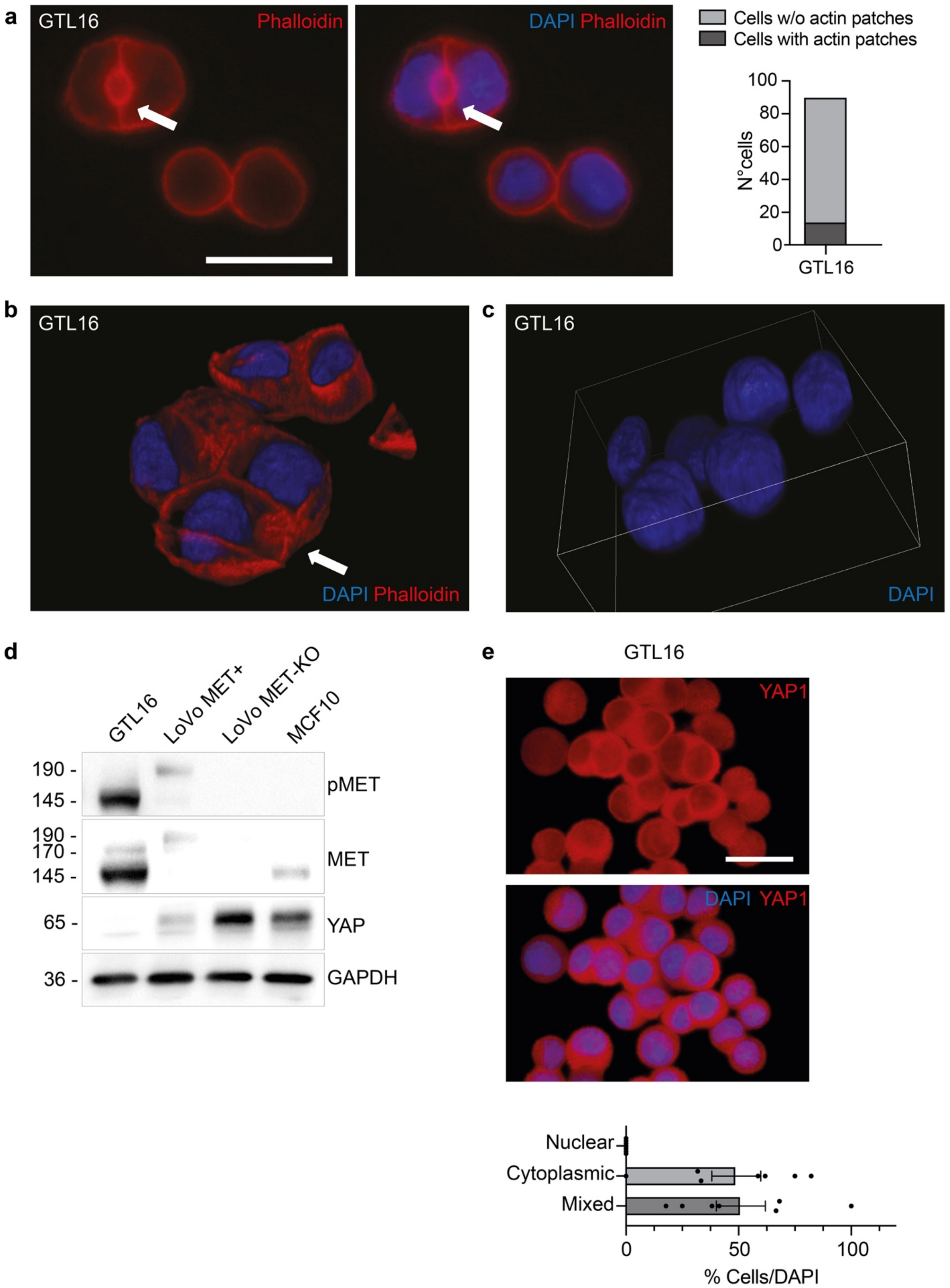

Supp. Figure 2: The gastric cell line GTL16 display actin aberrations, spherical nuclei and low YAP1 activation. a) GTL16 cells stained with Phalloidin (red) and counterstained with DAPI (blue). White arrows indicate the actin patches. Scale bar: 25µm. A quantification of cells with and without an actin patch is provided. A total number of 94 cells was analyzed in 9 independent fields. **b, c)** Confocal 3D rendering of representative GTL16 cells stained with Phalloidin (red) and DAPI

(blue) (b) and DAPI alone (c). The white arrow indicates the actin patch. **d)** Evaluation of MET and YAP1 protein levels in a panel including the four cell models employed in the present study. GAPDH was used as loading control. **e)** Analysis of YAP1 subcellular localization through immunofluorescence. Cells were stained with YAP1 antibody (red) and counterstained with DAPI (blue). Quantification is provided below. 183 cells were analyzed. Scale bar: 50μm.

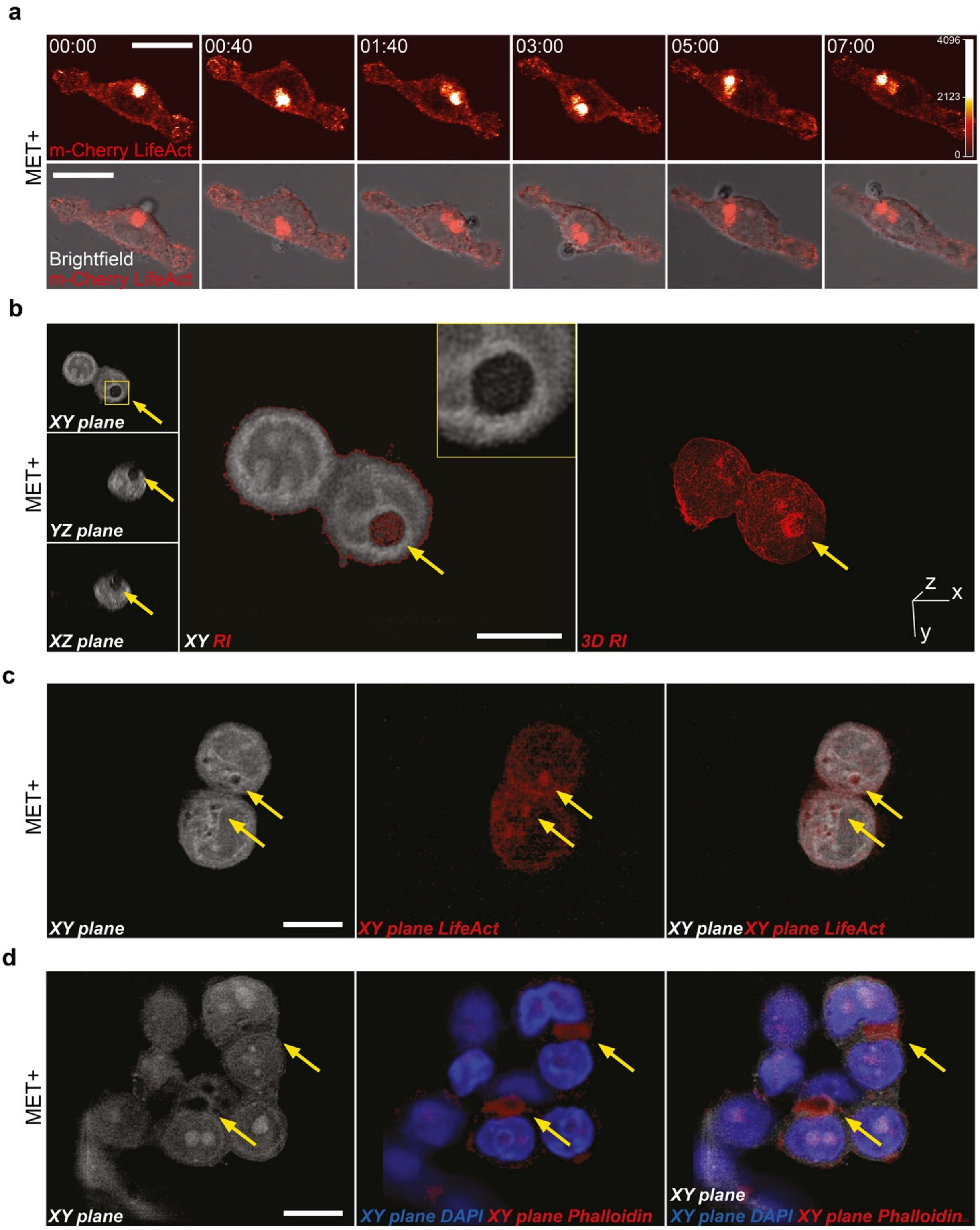

Supp. Figure 3: Actin aberrations of MET+ cells are visible in living cells with LifeAct and holotomography. a) Time-lapse of mCherry-LifeAct LoVo MET+ cells monitored for 7 hours with confocal imaging. In the upper panel, a virtual LUT was applied to highlight the areas with intense red signal according to the scale on the right. In the lower panel, both brightfield and mCherry-LifeAct (red) signal are shown. Scale bar: 10µm. **b)** Holotomography and 3D reconstruction of living LoVo MET+ cells. XY, YZ and XZ plane view are provided. Yellow arrows points at the actin patch. Scale bar: 10µm. **c)** Holotomography and mCherry-LifeAct (red) detection in LoVo MET+ cells. A merge of the two signal is provided to show the overlapping. Yellow arrows points

at the actin patch. Scale bar: 10μm. **d)** Holotomography and Phalloidin (red)/DAPI (blue) detection in fixed LoVo MET+ cells. A merge of the two signal is provided to show the overlapping. Yellow arrows points at the actin patch. Scale bar: 10μm.

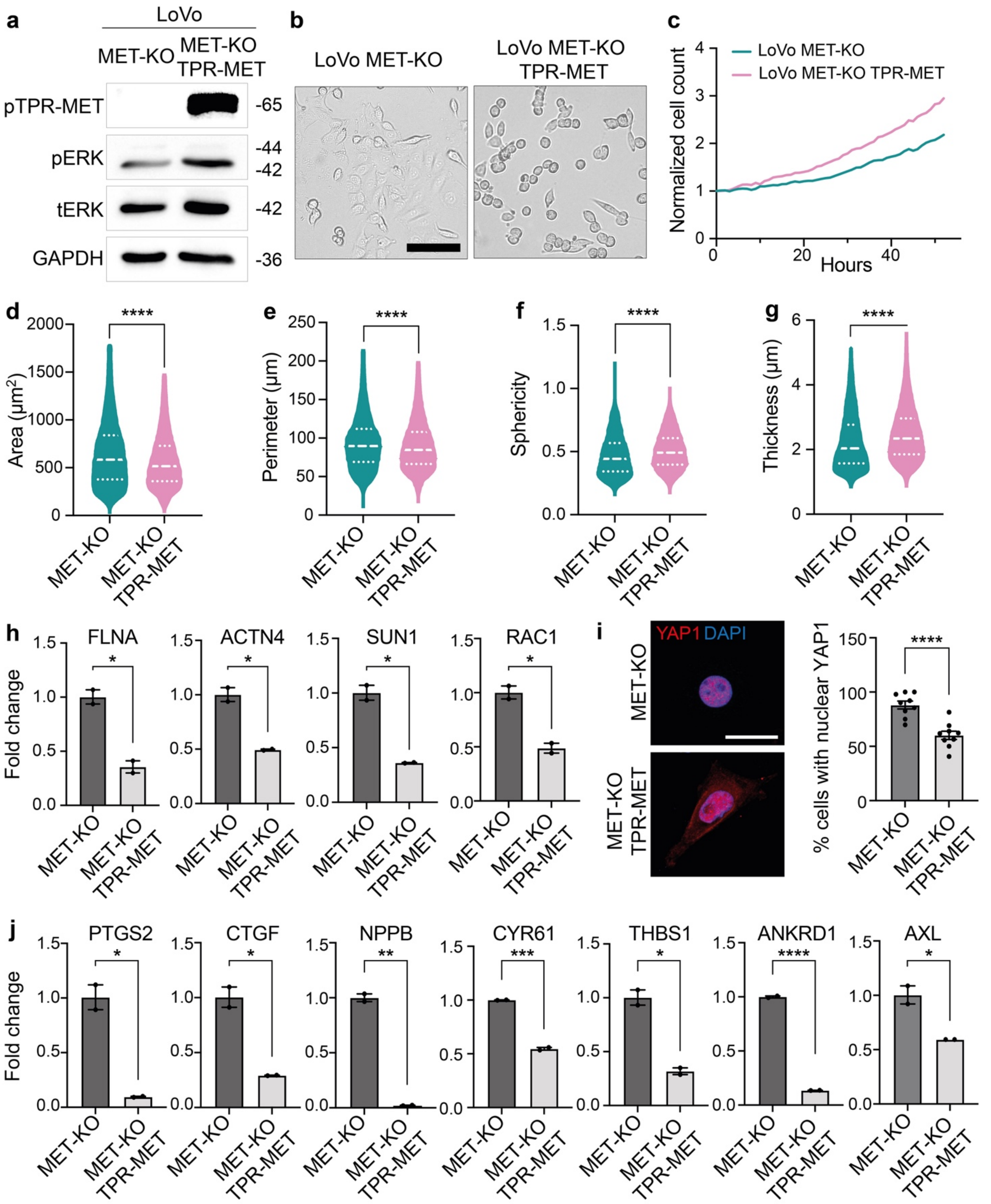

Supp. Figure 4: MET rescue in MET-KO LoVo cells through TPR-MET insertion reproduces the phenotype of LoVo MET+ cells. a) Western blot validation of TPR-MET introduction in MET-KO cells. GAPDH was used as loading control. **b)** Micrographs of LoVo MET-KO and MET-KO TPR-MET cells in culture. Scale bar: 50μm. **c, d, e, f, g)** Quantification of cell count, area, perimeter, sphericity and thickness in MET-KO and MET-KO TPR-MET cells monitored for 55 hours through Phasefocus Livecyte^{TM} platform. Pictures were taken every 2 hours. Median and quartiles distribution are plotted. 1500 cells were analyzed. Outliers were identified and cleaned from results by means of ROUT method (Q = 1%). Statistic was calculated by unpaired T-test.

h) Analysis of actin cap-related genes FLNA, ACTN4, RAC1 and SUN1 RNA expression by qPCR in MET-KO and MET-KO TPR-MET cells. Data are shown as fold change in relation to MET-KO ± SD. Unpaired T-test was applied for statistical analysis. **i)** Evaluation of YAP1 subcellular localization in MET-KO and MET-KO TPR-MET cells. Cells were counterstained with DAPI (blue). Scale bar: 25μm. The % of cells with complete nuclear YAP1 is reported in the quantification. A total of 493 (MET-KO) and 413 (MET-KO TPR-MET) cells were analyzed from pictures taken in 9 independent fields. Data are shown as mean ± SEM. Statistic was calculated by T-test. Scale bar: 50μm. **j)** Assessment of mRNA levels of 7 YAP1 target genes (PTGS2, CTGF, NPPB, CYR61, THBS1, ANKRD1, AXL) in MET-KO and MET-KO TPR-MET cells by qPCR. Data are shown as fold change in relation to MET-KO ± SD. Unpaired T-test was applied for statistical analysis.

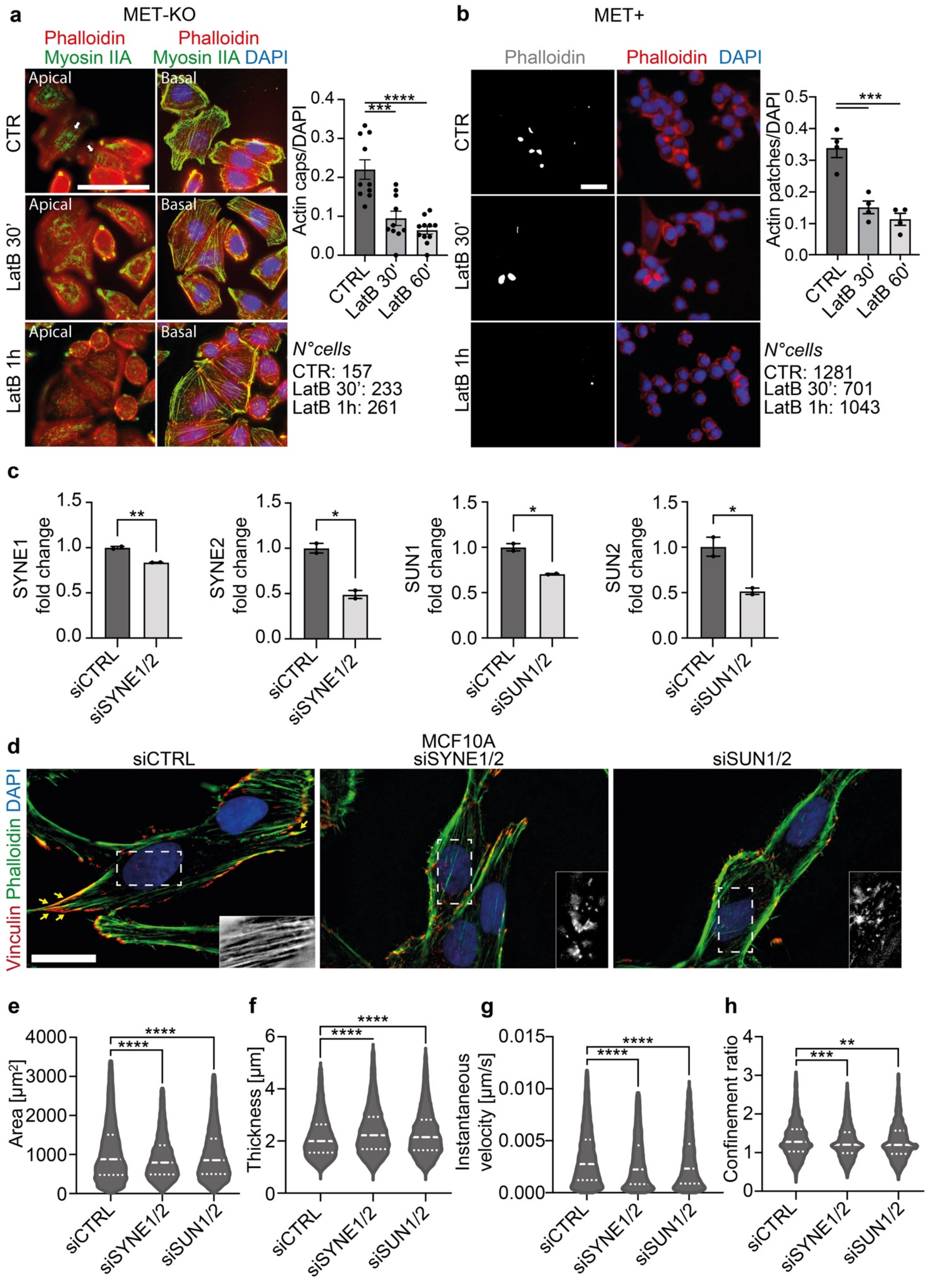

Supp. Figure 5: Low doses of Latrunculin B and siRNA against LINC complex components affect actin cap formation and global cell phenotype. a) Phalloidin (red), Myosin IIA (green) and DAPI (blue) staining in LoVo MET-KO cells treated with Latrunculin B 80nM for 30' and 1h. A

quantification of the number of cells with properly organized actin cap is provided. Data are reported as mean ± SEM. The number of analyzed cells is reported in the figure. Statistics was calculated by One-way ANOVA. Scale bar: 50µm **b)** Phalloidin (red) and DAPI (blue) staining in LoVo MET+ cells treated with Latrunculin B 80nM for 30' and 1h. A quantification of the number of cells with aberrant actin patches is provided. Data are reported as mean ± SEM. Statistics was calculated by One-way ANOVA. Scale bar: 25µm. **c)** qPCR validation of SYNE1/2 and SUN1/2 RNA interference by siRNA in MCF10A cells. Unpaired T-test was applied for statistical analysis. **d)** Phalloidin (green), DAPI (blue) and vinculin (red) staining of MCF10A cells transfected with siSYNE1/2 and siSUN1/2. Details show the apical plane of section of cells, where the actin cap fibers are located. Yellow arrows point at the actin cap associated focal adhesions (ACAFAs). Scale bar: 25µm. **e, f, g, h)** Quantification of cell area, thickness, instantaneous velocity and confinement ratio in MCF10A and MCF10A TPR-MET cells transfected with siSYNE1/2 or siSUN1/2 and monitored for 50 hours through Phasefocus Livecyte^{TM} platform. Pictures were taken every hour. Median and quartiles distribution are plotted. 1500 cells were analyzed. Outliers were identified and cleaned from results by means of ROUT method (Q = 1%) and T-test was applied for statistical analysis.

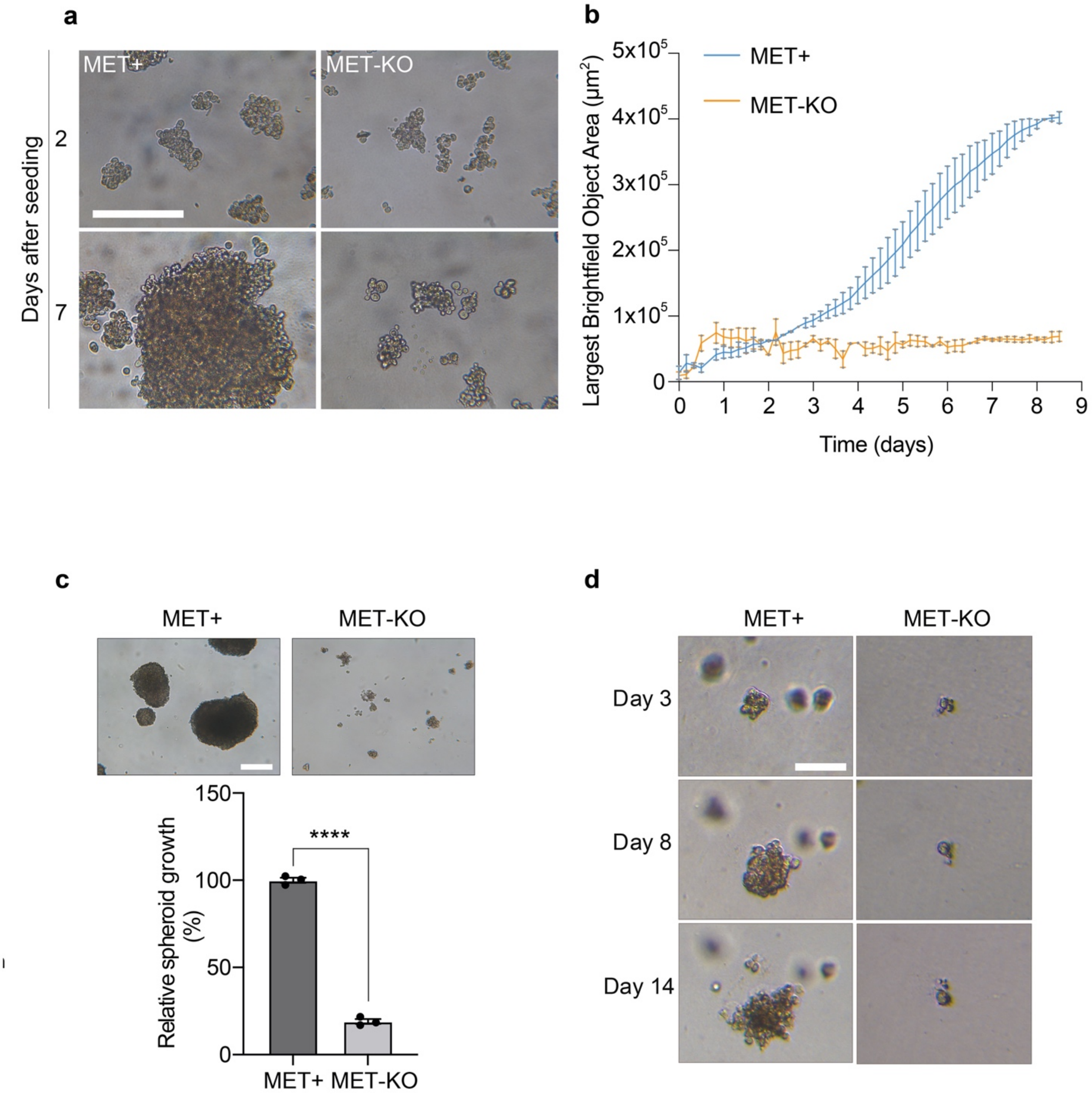

Supp. Figure 6: MET silencing totally impairs LoVo cells capability to proliferate in lack of adhesion and to grow in matrigel. a, b) Representative pictures and quantification showing LoVo MET+ and MET-KO cells grown in suspension. Cells were seeded in 96-wells over a layer of Agar 0,6% to prevent cell adhesion. Spheroids formation was monitored with IncuCyte platform for a total of 9 days. The quantification of the area of the largest spheroid identified by IncuCyte software is provided. Scale bar: 250µm. **c)** Quantification of MET+ and MET-KO spheroids proliferation by means of AlamarBlue (resazurin) assay. Cells grown for 15 days as in (A) were incubated for 4 hours with AlamarBlue reagent before detecting signal output. Data are reported as mean (%) ± SEM related to MET+ values. Statistics was calculated by T test. Scale bar: 500µm **d)** Representative pictures showing LoVo MET+ and MET-KO growing embedded in matrigel for two weeks. Scale bar: 100µm.

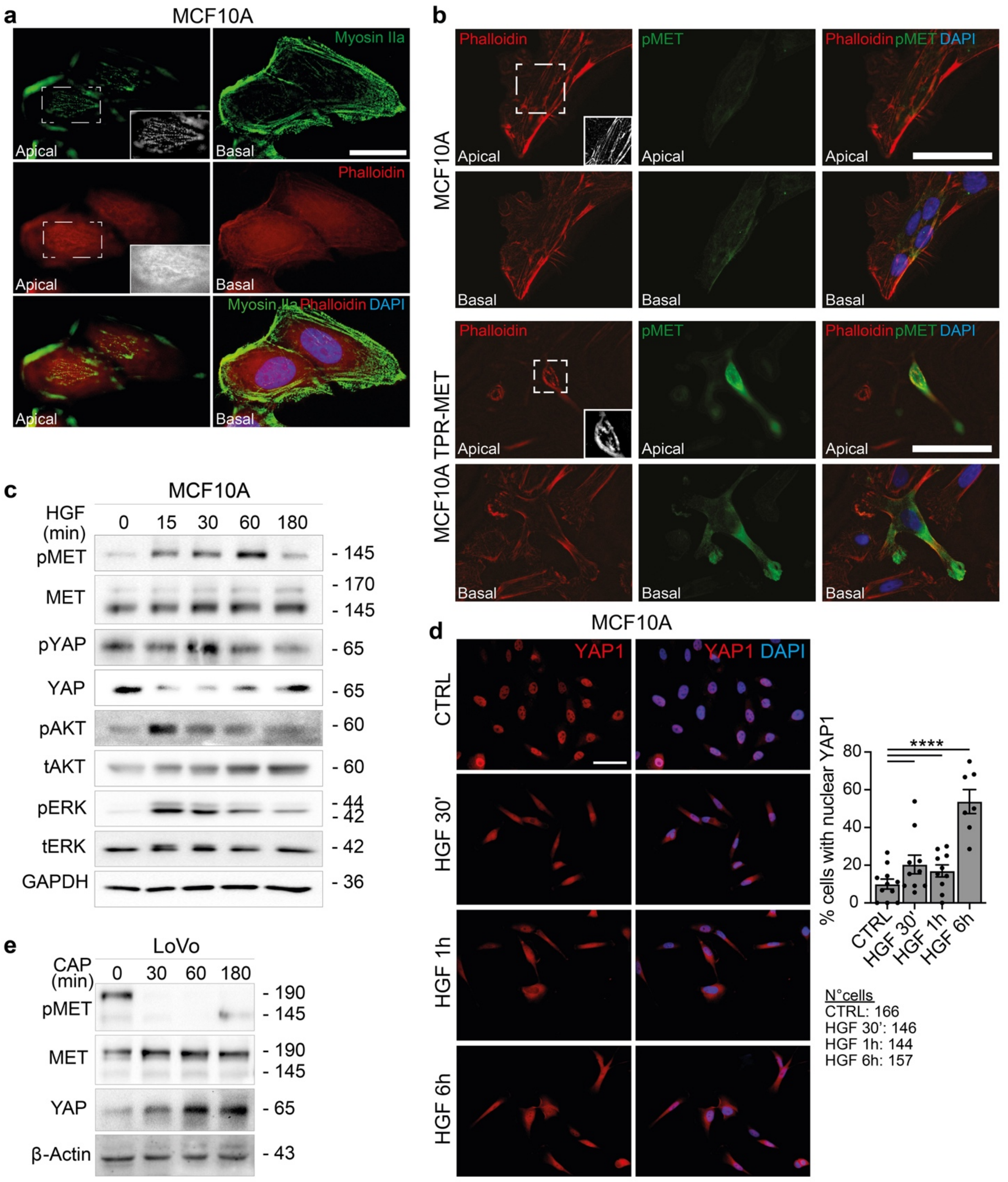

Supp. Figure 7: YAP1 pathway is inhibited by MET activation and boosted by MET interception. a) Myosin IIA (green), Phalloidin (red) and DAPI (blue) staining in MCF10A cells highlighting the structure of the perinuclear actin cap (apical section) and basal stress fibers (basal section). Scale bar: 25µm. **b)** pMET (green), Phalloidin (red) and DAPI (blue) staining in MCF10A WT and TPR-MET. Representative pictures show both apical and basal plane. Scale bar: 50µm. **c)** Analysis of YAP and MET activation, as well as AKT and ERK phosphorylation in MCF10A cells treated with HGF 50ng/mL over a 3 hours-time course. GAPDH was employed as loading control. **d)** Evaluation of YAP1 (red) subcellular localization in MCF10A cells treated with HGF 50ng/mL for 30', 1h and 6h. Cells were counterstained with DAPI. Scale bar: 50µm. A quantification of %

cells with nuclear YAP1 is provided on the right, and the number of analyzed cells is reported. Data are shown as mean (%) ± SEM and statistics was calculated by one-way ANOVA. **e)** Analysis of YAP protein levels upon capmatinib 1μM administration for 30'-1h-3h in LoVo cells. β-actin was used as loading control.

Figure 1a

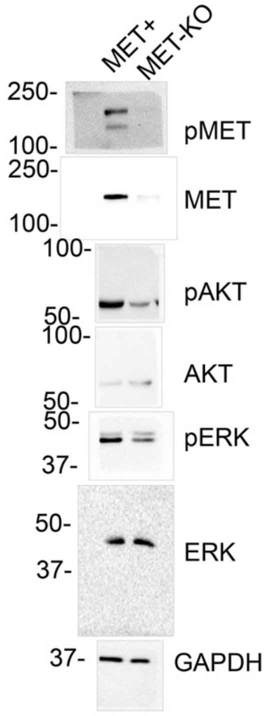

Figure 5e

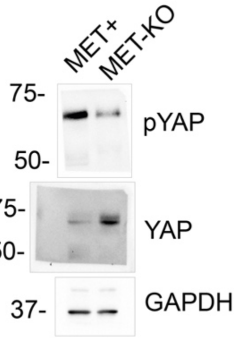

Figure 6a

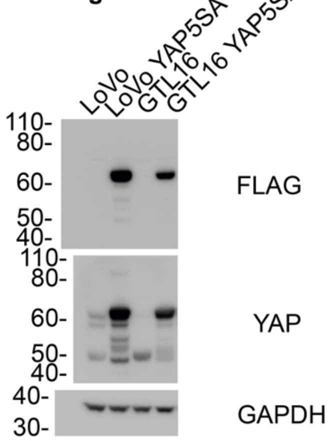

Figure 7a

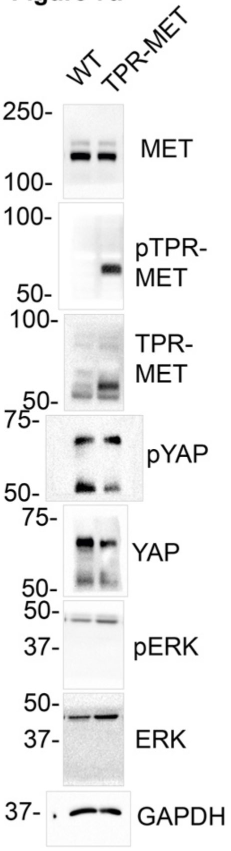

Supp. Figure 4a

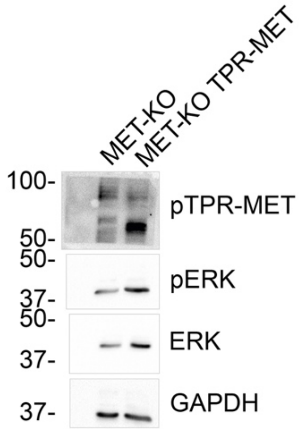

Supp. Figure 7c

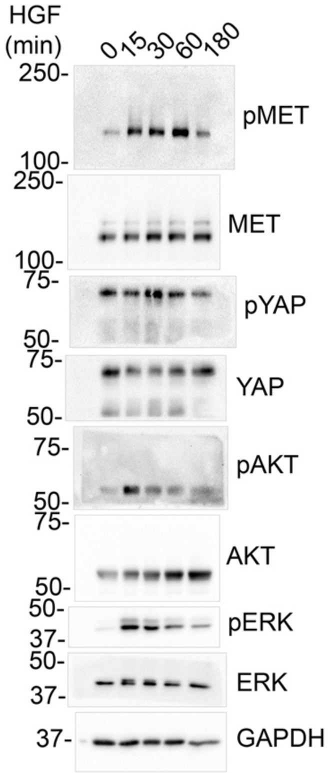

Supp. Figure 7e

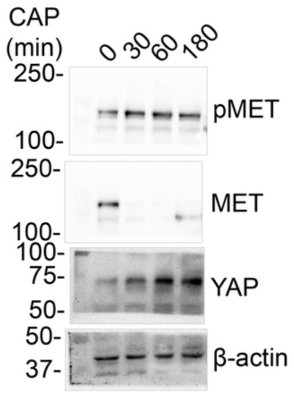

Supp. Figure 8: Original uncropped blots from Fig.1a, 5e, 7a, S4a, S7c and S7e.
