# Supplementary material for: Aberrant MET activation impairs perinuclear actin cap organization with YAP1 cytosolic relocation
Source: Commun Biol. 2023 Oct 14;6:1044. doi: 10.1038/s42003-023-05411-y (PMC10576810; doi:10.1038/s42003-023-05411-y)
Supplement: Supplementary file 2 — Description of Additional Supplementary Files [file 42003_2023_5411_MOESM2_ESM.pdf]

Description of Additional Supplementary Files

File name: Supplementary Data 1
Description: The source data behind the graphs in the paper.

File name: Supplementary Video 1
Description: Confocal time-lapse of MET+ mCherry-LifeAct cells. Cells were monitored for a total of 16 hours and images were taken every 20 minutes. A virtual LUT was applied to highlight the areas with intense red signal according to the scale in Supp. Figure 2a.

File name: Supplementary Video 2
Description: Confocal time-lapse of MET+ mCherry-LifeAct cells. Cells were monitored for a total of 16 hours and images were taken every 20 minutes. A merge of mCherry-LifeAct and brightfield view is shown.

File name: Supplementary Video 3
Description: Time-lapse of MET+ cells performed with optical diffraction microscopy (ODT) imaging. Cells were monitored for a total of 12h. Images were taken every 10 minutes.

File name: Supplementary Video 4
Description: Time-lapse of MET+ cells performed with optical diffraction microscopy (ODT) imaging. Cells were monitored for a total of 3h. Images were taken every 10 minutes.

File name: Supplementary Video 5
Description: Time-lapse of MET-KO cells performed with optical diffraction microscopy (ODT) imaging. Cells were monitored for a total of 8h. Images were taken every 10 minutes.

File name: Supplementary Video 6
Description: Time-lapse of MET+ cells performed with Phasefocus LivecyteTM. MET+ cells were seeded in 96-wells plates and incubated for 12h before starting image acquisition. Cells were monitored for more than 48h, and images were taken every 1-4h.

File name: Supplementary Video 7
Description: Time-lapse of MET-KO cells performed with Phasefocus LivecyteTM. MET-KO cells were seeded in 96-wells plates and incubated for 12h before starting image acquisition. Cells were monitored for more than 48h, and images were taken every 1-4h.

File name: Supplementary Video 8
Description: Time-lapse of MET-KO TPR-MET cells performed with Phasefocus LivecyteTM. MET-KO TPR-MET cells were seeded in 96-wells plates and incubated for 12h before starting image acquisition. Cells were monitored for more than 48h, and images were taken every 1-4h.

File name: Supplementary Video 9
Description: Time-lapse of MET+ cells grown in suspension performed with IncuCyte® platform. MET+ cells were seeded over a layer of 0.6% agar to prevent cell adhesion. Starting from the day after seeding, spheroids formation was monitored with IncuCyte® for 7 days and 16h, and images were acquired every 4h.

File name: Supplementary Video 10
Description: Time-lapse of MET-KO cells grown in suspension performed with IncuCyte® platform. MET-KO cells were seeded over a layer of 0.6% agar to prevent cell adhesion. Starting from the day after seeding, spheroids formation was monitored with IncuCyte® for 7 days and 16h, and images were acquired every 4h.
